# Downregulation of CD151 restricts VCAM-1 mediated leukocyte infiltration to reduce neurobiological injuries after experimental stroke

**DOI:** 10.1186/s12974-021-02171-6

**Published:** 2021-05-22

**Authors:** Ceshu Gao, Wangyue Jia, Wendeng Xu, Qiong Wu, Jian Wu

**Affiliations:** 1 Department of Neurology, Beijing Tsinghua Changgung Hospital, School of Clinical Medicine, Tsinghua University, Beijing, 102218 China; 2 MOE Key Laboratory of Bioinformatics, School of Life Sciences, Tsinghua University, Beijing, 100084 China

**Keywords:** CD151, VCAM-1, Endothelial cell, Neutrophil, Monocyte, Stroke

## Abstract

**Background:**

Translational failures in anti-adhesion molecule therapies after stroke reveal the necessity of developing new strategies that not only interrupt leukocyte recruitment but also consider the inhibition of endothelial cell inflammation, verification of therapeutic time window, and normal function maintenance of circulating leukocytes. Our study focused on the potential therapeutic value of CD151 downregulation in improving current anti-adhesion molecule therapies.

**Methods:**

Lentivirus intracerebroventricular administration was conducted to inhibit the CD151 expression and observe its functional influence on neurological injuries and outcomes. Then, immunohistochemistry and myeloperoxidase activity assessment were performed to explore the effects of CD151 expression on neutrophil and monocyte recruitment after rat cerebral ischemia. Primary rat brain microvascular endothelial cells were subjected to oxygen glucose deprivation and reoxygenation to elucidate the underlying working mechanisms between CD151 and VCAM-1.

**Results:**

The CD151 downregulation remarkably reduced neurological injuries and improved neurological outcomes, which were accompanied with reduced neutrophil and monocyte infiltration after the CD151 downregulation. The VCAM-1 expression was remarkably decreased among the adhesion molecules on the endothelial cell responsible for neutrophil and monocyte infiltration. The activation of p38 MAPK and NF-κB pathways was restricted after the CD151 downregulation. p38 MAPK and NF-κB inhibitors decreased the VCAM-1 expression, and p38 acted as an upstream regulator of NF-κB. However, CD151 downregulation did not directly influence the neutrophil and monocyte activation.

**Conclusions:**

Overall, CD151 regulated the expression of adhesion molecules. It also played a critical role in suppressing VCAM-1-mediated neutrophil and monocyte infiltration via the p38/NF-κB pathway. This study possibly provided a new basis for improving current anti-adhesion molecule therapies.

**Supplementary Information:**

The online version contains supplementary material available at 10.1186/s12974-021-02171-6.

## Background

Leukocyte infiltration after cerebral ischemia is linked to secondary injury after ischemic stroke [1]. It is also associated with a poor prognosis in clinical trials and experimental animal studies [2, 3]. Neutrophils and monocytes are among the major immune cells that infiltrate after cerebral ischemia and their infiltration peaks within 3 days after cerebral ischemia [4]. As the base of leukocyte infiltration, the blockade of adhesion molecules responsible for the leukocyte-endothelial cell interaction has led to promising neurological outcomes [5]. However, anti-adhesion molecule therapies fail to meet the clinical needs. The translational failures of these therapies indicate the need for the development of reliable potential targets and the verification of optimal therapeutic time window [6].

New evidence suggests the emerging role of tetraspanin in the interaction of immune and endothelial cells. As a member of the tetraspanin family, CD151 is known as a modulator of the activities of different transmembrane protein families; it also functions as a partner of adhesion molecules during chronic inflammation [7]. CD151, CD9, VCAM-1, and ICAM-1 form tetraspanin-enriched microdomains (TEMs) [8]. Activated endothelial cells actually assemble preexisting TEMs to form endothelial docking structures instead of relying on the unstructured interaction of adhesion molecules and ligands [9, 10]. Thus, adhesion molecules (ICAM-1, VCAM-1, and E-selectin) respond to ligands presented by leukocytes and are regulated by CD151 on endothelial cells in the early stage. Therefore, the involvement of CD151 in adhesion molecule regulation enriches our understanding of the interaction between endothelial cells and leukocytes and may further explain the current failures in anti-adhesion molecule therapies. However, studies have yet to clarify whether the role of tetraspanin, especially CD151, in TEMs is valuable enough to bridge the translational gap in terms of anti-adhesion molecule therapies in ischemic stroke.

This work aimed to investigate the therapeutic value of CD151 downregulation and its function in leukocyte infiltration after cerebral ischemia. Herein, we specifically focused on the changes in the expression of TEM components and other adhesion molecules in endothelial cells in neutrophil and monocyte recruitment after experimental stroke.

## Methods

### Animals

A total of 239 specific pathogen-free male Sprague-Dawley rats were included, and a mortality of around 6% was observed. The rats weighing 250–260 g were first purchased from the Beijing Vital River Laboratory Animal Technology Co. Experimental protocols were approved by the Institutional Animal Care and Use Committee at Tsinghua University. The animals were placed in a house with a 12-h light/dark cycle condition, a temperature of 23 °C ± 3 °C, a relative humidity of 50–60%, and ad libitum access to water and chow. All the rats were randomly assigned to different groups. All the experiments followed a double-blind method.

### Middle cerebral artery occlusion (MCAO) model in rats

A rat experimental ischemic stroke model was established as previously described [11]. After lentivirus intracerebroventricular administration and 7-day normal breeding, the weight of rats naturally increased. The adult rats (295–305 g) were initially anesthetized with 5% isoflurane and maintained with 2–2.5% isoflurane during surgery. The body temperature of the rats was maintained at 37 °C ± 0.5 °C by using a surface heating pad. Experimental cerebral ischemia was induced by inserting a silicone-coated filament (Cinontech, China) into the right common carotid artery to block the middle cerebral artery (MCA). After 2 h MCAO, the filament was withdrawn to restore the blood flow. Sham-operated rats were subjected to the same procedure, but the filament was inserted into the carotid artery without blocking the MCA.

### Lentivirus construction, intracerebroventricular (ICV) administration, and effectiveness measurement

The lentivirus for expressing the CD151 shRNA (LV CD151 shRNA) and the scramble lentivirus with an inert random shRNA sequence (LV Vehicle) were purchased from GenePharma Corporation (Shanghai, China). Both the two lentiviruses expressed GFP tag. The lentivirus shRNA information is presented in detail in Table S1. The effectiveness of different modes of lentivirus CD151 shRNA transfection was confirmed through Western blot and RT-PCR (Additional file: Figure S1).

The rats were divided into three groups, namely, lentivirus vehicle injection and sham surgery (LV Vehicle + Sham), lentivirus vehicle injection and MCAO (LV Vehicle + MCAO), and lentivirus CD151 shRNA injection and MCAO (LV CD151 shRNA + MCAO).

The rats were transfected with the lentivirus through ICV injection 7 days before the MCAO surgery. Briefly, they were anesthetized with 2% sodium pentobarbital (60–80 mg/kg) and placed in a stereotaxic apparatus (Cinontech, China). The ICV injection was performed using a 10-μl syringe, and coordinates were 2 mm lateral to the bregma, 1.5 mm posterior to the bregma, and 4–5 mm deep from the dura. The rats received either 8 μl of LV CD151 shRNA (1 × 10^9^ TU/ml) or 8 μl of LV vehicle (1 × 10^9^ TU/ml) at a rate of 0.5 μl/min. After 5 min, the needle was slowly withdrawn to prevent reflux.

**Fig. 1:**
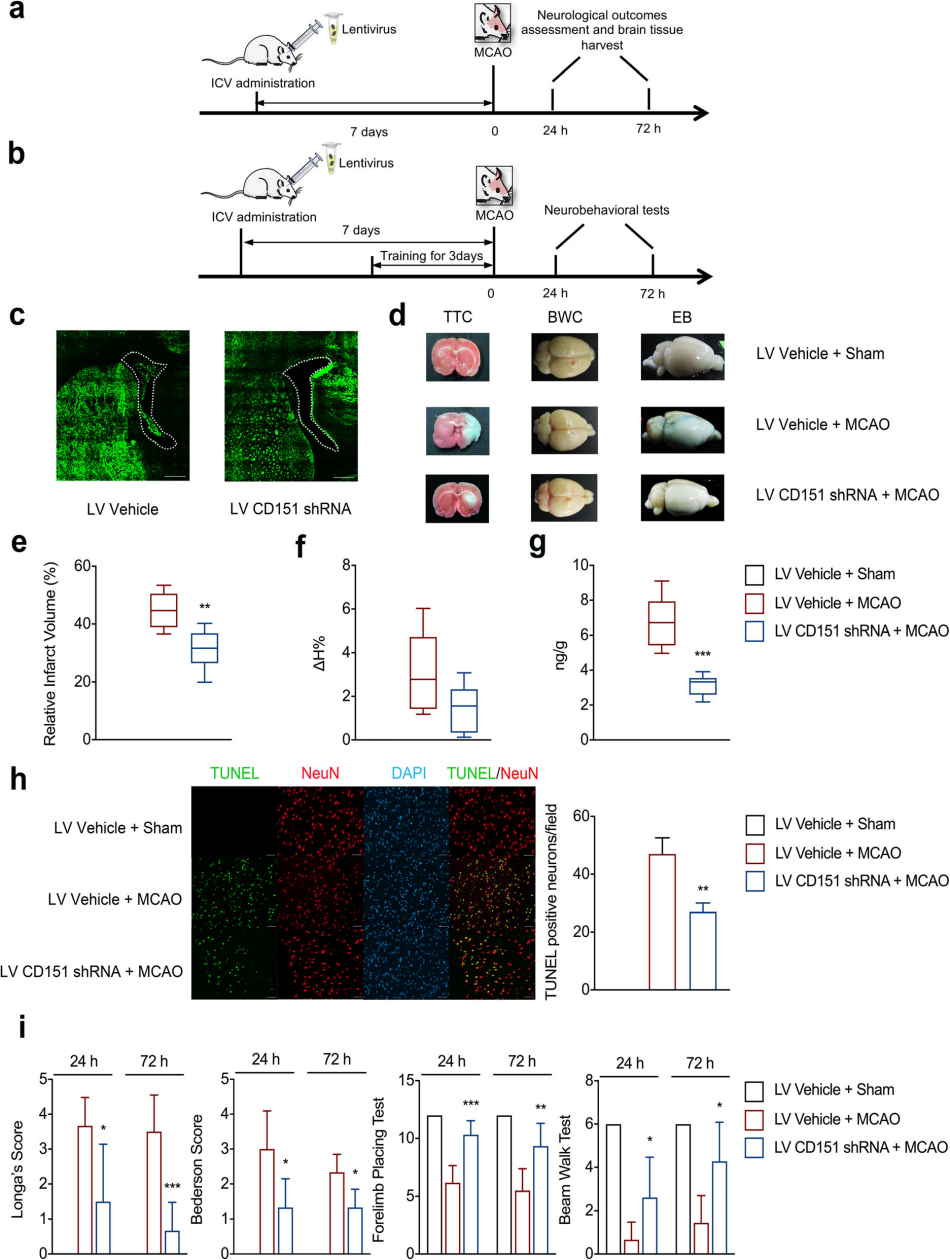
In vivo experimental results suggested that the CD151 downregulation had a protective function after experimental stroke. **a**, **b** In vivo experiment design. Rats were subjected to LV shCD151/LV vehicle subventricular injection 7 days prior to 2-h middle cerebral artery occlusion ischemia surgery. After 24- or 72-h reperfusion, rats underwent **a** sacrifice or **b** behavioral test. **c** Successful lentivirus transfection was detected by GFP expression in brain cryosections both in the LV vehicle and LV CD151 shRNA groups; the white dotted line showed the right lateral ventricles (scale bar = 500 μm). **d** Rat’s brain tissue for infarct volume (TTC), brain water content (BWC), and Evan’s blue (EB) measurements. **e** Infarction volume measured using TTC (*n* = 6 per group). ** vs. LV Vehicle + MCAO indicates *p* < 0.01. **f** BWC 24 h after reperfusion (*n* = 6 per group, no significant difference was found). **g** EB test for the evaluation of BBB degradation (*n* = 6 per group). *** vs. LV Vehicle + MCAO indicates *p* < 0.001. **h** Neuronal apoptosis at 24 h after reperfusion (*n* = 3 per group, scale bar = 100 μm). ** vs. LV Vehicle + MCAO indicate *p* < 0.01. **i** Neurobehavioral tests, including Longa’s score, Bederson score, forelimb placing test, beam walk test, 24 h and 72 h after reperfusion (*n* = 6 per group). *, **, and *** vs. LV Vehicle + MCAO indicate *p* < 0.05, 0.01, and 0.001, respectively

The GFP expression, which indicated successful lentivirus infection in vivo was detected with LSM880 Airyscan (Zeiss, Germany) in the cryosections of the brain tissues 7 days after lentivirus injection (Fig. 1c).

### Infarct volume measurement

The rats were sacrificed 24 h after reperfusion. As previously described [12], the brains were harvested and cut into slices with 2-mm thickness. Six pieces of each brain were incubated at 37 °C in a 2,3,5-triphenyltetrazolium chloride solution (Servicebio, China) for 20 min and in 4% PFA for 24 h. The relative infarct volume was analyzed using the image analysis software (ImageJ) and expressed as a ratio to the contralateral hemisphere to determine the whole infarct volume of each brain.

### Brain water content measurement

The ischemic and contralateral hemispheres were collected 24 h after reperfusion [13]. The wet weight right after the brain was harvested, and the dried weight, which was the tissue weight after treatment at 110 °C for 24 h, was recorded. The relative brain water content was calculated using the weight change difference between the two hemispheres.

### Evans blue extravasation

The rats were injected with Evans blue (2% in saline, 3 ml/kg; Sigma, USA) injection through the tail vein 1 h before they were sacrificed. Before the brain tissue was harvested, the rats were perfused with phosphate-buffered saline (PBS) to remove the circulating dye. Then, the brain tissue was homogenized in 50% trichloroacetic acid (2 ml), and the homogenate was centrifuged at 13,000*g* for 10 min. The supernatant (25 μl) was mixed with ethanol (100 μl), and the absorbance at 632 nm was measured using a spectrophotometer (Thermo Fisher Scientific, USA). The content of Evans blue was quantified using a standard curve and expressed in nanograms of Evans blue per gram of brain tissue.

### Neurobehavioral tests

Neurobehavioral tests, including Longa’s score, Bederson score, forelimb placing test, and beam walking test, were conducted 24 and 72 h after reperfusion. All the assessments were performed by an investigator blinded to the group treatment.

Longa’s scoring system (0, no deficit; 1, failure to extend the left forepaw; 2, circling to the left; 3, falling to the left; 4, failure to walk spontaneously and loss of consciousness; and 5, death) was adopted to evaluate neurological deficits [11].

The Bederson score (0, no deficit; 1, forelimb flexion; 2, forelimb flexion and decreased resistance to lateral push; 3, unidirectional circling; 4, longitudinal spinning or seizure activity; and 5, no movement) was used to evaluate the overall neurological function [14].

A forelimb placing test was carried out to assess the response to sense tactile stimulation from the vibrissae and the eyes. The test was composed of four parts. First, the rats were held gently by the tail, and their heads were slowly lowered to the table surface. Normally, the rats stretched out their forelimbs to the surface with symmetrical movements in the air. Second, the rats’ forelimbs were placed at the edge of the table and gently pushed against the edge. Normally, the rats successfully resisted pushing. Third, the rats were placed parallel to the table edge, and their forelimbs were forced to the edge. Normally, the rats withdrew their paws immediately. Fourth, the rats’ hindlimbs were placed on the table edge, and each paw was taken down alternately. Normally, the rats repositioned their legs quickly. Scores were recorded as follows: normal performance, 2 points; delayed (> 2 s) and/or incomplete performance, 1 point; and no performance, 0 points [15].

The beam walk test was used to evaluate motor coordination. The rats were first trained for 3 days before MCAO surgery and pre-tested to pass through the beam (90 cm long, 2.5 cm wide, and 80 cm high) voluntarily without a slip. Motor performance was scored as follows: 0, no attempt to stay on the beam; 1, attempted to stay on beam but no movement; 2, attempted to cross the beam but failed; 3, crossed the beam but the contralateral hindlimb slipped by > 50%; 5, crossed the beam but the contralateral hindlimb slipped by < 50%; and 6, crossed the beam without a slip [16].

### Neuronal apoptosis assessment

Three 15-μm-thick brain tissue cryosections (position: bregma + 2 mm, bregma 0 mm, and bregma − 2 mm) from each brain were obtained 24 h after reperfusion. Terminal deoxynucleotidyl transferase dUTP nick end labeling (TUNEL) and NeuN labeling (1:50, Cell Signaling Technology, USA) were processed for neuronal apoptosis analysis in accordance with the manufacturer’s instructions (Roche, USA). Immunofluorescence images were captured using Axio Scan.Z1 (Zeiss, Germany). For each section, the average number of TUNEL-positive neurons was calculated from six randomly chosen fields from the penumbra. The average number of the three sections was recorded as the neuronal apoptosis value for each brain.

### Immunohistochemistry

Three 8-μm-thick brain tissue cryosections (position: bregma + 2 mm, bregma 0 mm, and bregma − 2 mm) from each brain were collected 24 or 72 h after reperfusion. Myeloperoxidase (MPO) and CD115 were used as biomarkers to identify infiltrating neutrophils and monocytes, respectively. Endogenous peroxidase was blocked with a peroxidase blocking solution (Servicebio, China) for 30 min after 4% PFA fixation. Then, all the sections were washed with PBS containing 0.1% Tween-20 (Solarbio, China) and incubated with the primary antibody of MPO (1:500, Abcam Cambridge, UK) or CD115 (1:50, Santa Cruz, USA) for 60 min at 21 °C and with the secondary antibody for 60 min. An immunoenzyme polymer (Servicebio, China) was used for MPO or CD115 and developed in diaminobenzidine to visualize immunoreactivity. Immunohistochemistry images were collected using Pannoramic SCAN (3DHISTECH, Hungary). For each section, the average number of MPO-positive cells or CD115-positive cells was calculated from six randomly chosen fields from the penumbra. The average of the three sections was recorded as infiltrating neutrophils for each brain.

### MPO activity assessment

A commercially available MPO activity kit (Genmed Scientific Inc., USA) was used to reveal neutrophil activation. The ischemic hemisphere was harvested at 24 or 72 h after reperfusion, and MPO assessment was performed in accordance with the manufacturer’s instructions. Briefly, the total protein was extracted using the reagents in the kit, and the absorbance at 645 nm was measured using spectrophotometer (Thermo Fisher Scientific, USA). The results were presented as units per gram of the brain.

### Primary rats’ brain microvascular endothelial cell (BMVEC) culture, lentivirus transfection, and oxygen glucose deprivation (OGD)

The primary rats’ BMVECs were commercially purchased (Procell, China). The LV CD151 shRNA and the LV Vehicle used for in vitro transfection were the same as those used in the in vivo experiments (Genepharma, China) at a multiplicity of infection of 50. Transfection efficiency was tested using the expression of a green fluorescent protein through immunofluorescence by Olympus IX81 (Olympus, Japan). The CD151 expression was determined using Western blot (Additional file: Figure S3 and S4). Four in vitro experimental groups, namely, only OGD, only lentivirus vehicle transfection (LV Vehicle), lentivirus vehicle transfection and OGD (LV Vehicle + OGD), and lentivirus CD151 shRNA and OGD (LV CD151 shRNA + OGD), were set. The cultured primary BMVECs were subjected to in vitro ischemia treatment 96 h after lentivirus transfection. For consistency with the in vivo experiments, 2 h of OGD, 95% N_2_, and 5% CO_2_, glucose-free Dulbecco’s modified Eagle medium, and reoxygenation for 24 h in a complete medium were performed.

### HL-60 culture and stimulation of the BMVEC supernatant

The HL-60 cell line was kindly donated by Dr. Yumin Luo from the Institute of Cerebrovascular Disease Research, Xuanwu Hospital of Capital Medical University. BMVECs were subjected to OGD and reoxygenation treatment, and the supernatant was collected. The same amounts of BMVEC supernatant and HL-60 suspension were mixed. After being cultured for 72 h, HL-60 as well as the supernatant were harvested for further measurement.

### THP-1 culture and stimulation of the BMVEC supernatant

The THP-1 cell line was kindly donated by Dr. Zhenglin Ji from the School of Medicine, Tsinghua University. BMVECs were subjected to OGD and reoxygenation treatment, and the supernatant was collected. The same amounts of BMVEC supernatant and THP-1 suspension were mixed. After being cultured for 72 h, THP-1 and the supernatant were harvested for further measurement.

### Protein extraction and Western blot

The M-PER mammalian protein extraction reagent (Thermo Fisher Scientific, USA) was used for total protein extraction from the brain tissue and cultured primary BMVECs. The NE-PER nuclear and cytoplasmic extraction reagents (Thermo Fisher Scientific, USA) were used for fresh cultured primary BMVECs, and the Minute™ Cytosolic and Nuclear Extraction Kit for Frozen/Fresh Tissues (Invent Biotechnologies Inc., USA) was utilized for frozen brain tissues. The protease and the phosphatase inhibitor cocktails (Thermo Fisher Scientific, USA) were added to the protein extraction steps. All the procedures were conducted in accordance with the manufacturer’s instructions.

Protein samples (20 μg) were separated through 8–12% sodium dodecyl sulfate-polyacrylamide gel electrophoresis (SDS-PAGE) and transferred onto PVDF membranes. The membranes were blocked with the NcmBlot blocking buffer (NCM Biotech, China) for 10–20 min at room temperature, stripped with the NCM Western blot stripping buffer (NCM Biotech, China), and incubated overnight at 4 °C with the following primary antibodies: anti-CD151(1:500, Bioss, China), VCAM-1 (1:1000, Santa Cruz, USA), ICAM-1 (1:5000, Abcam Cambridge, UK), E-selectin (1:500, Proteintech, USA), CD9 (1:1000, Proteintech, USA), p-p38 (1:1000, Cell Signaling Technology, USA), p38 mitogen-activated protein kinase (p38; 1:1000, Proteintech, USA), p-JNK (1:1000, Cell Signaling Technology, USA), Jun N-terminal kinase (JNK; 1:3000, Proteintech, USA), p-ERK (1:2000, Cell Signaling Technology, USA), extracellular-related protein kinases (ERK; 1:1000, Proteintech, USA), nuclear factor kappa-B (NF-κB) inhibitor α (IκB α; 1:1000, Proteintech, USA), p65 (1:1000, Proteintech, USA), YY1 (1:5000, Proteintech, USA), VLA4 (1:1000, Proteintech, USA), LFA1 (1:500, Proteintech, USA), CD44 (1:2000, Proteintech, USA), PSGL1 (1:500, Proteintech, USA), β-actin (1:4000, Proteintech, USA), and glyceraldehyde-3-phosphate dehydrogenase (GAPDH; 1:5000, Proteintech, USA). The membranes were incubated with secondary antibodies (goat antirabbit IgG or goat antimouse IgG, 1:2000, Proteintech, USA) and visualized using an enhanced chemiluminescent substrate (Millipore, USA). The optical densities of the bands were scanned and quantified using image analysis systems (LAS-4000, GE, USA). β-actin, GAPDH, and YY1 were set as internal controls.

### Assay of intracellular reactive oxygen species (ROS)

ROS in HL-60 and THP-1 cultured with BMVEC supernatant for 72 h were measured with a ROS assay kit (Keygen Biotech, China). All the steps were performed in accordance with the manufacturer’s instructions. Briefly, the culture medium was first removed, and the cells were washed with PBS. Dihydroethidium (DHE), diluted with RPMI-1640 to a final concentration of 10 μM, was applied to resuspend the cells that were then incubated at 37 °C for 40 min. Fluorescence was read with a spectrophotometry (Thermo Fisher Scientific, USA) at 518 nm for excitation and 605 nm for emission.

### Enzyme-linked immunosorbent assay (ELISA)

A human neutrophil extracellular traps (NETs) ELISA kit (Meibiao Biology, China) was used to test the NET concentration in HL-60 supernatant after 72 h of culture with BMVEC supernatant. A human sCD14 ELISA kit and a human sCD163 ELISA kit (Multi Sciences, China) were used to detect THP-1 activation after 72 h of culture with BMVEC supernatant. All the steps were conducted in accordance with the manufacturer’s instructions.

### RT-PCR

The RT-PCR procedure is presented in detail in the Additional file.

### Statistical analysis

All data were presented as mean ± SD and analyzed using GraphPad Prism 8 (GraphPad Prism, USA). One-way ANOVA or unpaired t test was applied to determine the significance of differences among different groups. Tukey’s test was used for multiple comparison. *P* < 0.05 was considered significant.

## Results

### CD151 downregulation protected the brain from experimental ischemic stroke

Neurological outcomes were examined at 24 and 72 h after reperfusion to investigate the effect of the CD151 downregulation on experimental stroke outcomes in rats. The overall design of the in vivo experiments is shown in Fig. 1a, b. The effectiveness of lentivirus transfection was confirmed with immunofluorescence (Fig. 1c) and measured with Western blot and RT-PCR (Additional file: Figures S2 and S4).

The infarct volume of the LV CD151 shRNA + MCAO group was significantly smaller than that of the LV Vehicle + MCAO group (Fig. 1d, e). The Evan’s blue extravasation in the LV CD151 shRNA + MCAO group was also significantly lower than that in the LV Vehicle + MCAO group (Fig. 1d, g). Further evaluation of neuronal apoptosis at 24 h after reperfusion indicated that the LV Vehicle + MCAO group suffered a larger number of neuronal apoptosis than that of the LV CD151 shRNA + MCAO group (Fig. 1 h). Moreover, four behavioral tests (Fig. 1i), namely, Longa’s score, modified Bederson score, forelimb placing test, and beam walk test, consistently showed that the rats in the LV CD151 shRNA + MCAO group had better overall neurobehavioral outcomes at 24 and 72 h after reperfusion than that in the other groups. The brain water content in the LV CD151 shRNA + MCAO group decreased, but no statistical significance was addressed (Fig. 1d, f). Overall, the CD151 downregulation evidently protected the brain from ischemic injury.

### Reduced neutrophil and monocyte infiltration was partially due to the neuroprotective role of the CD151 downregulation

As the major player among immune cells that can migrate from circulation to the brain after stroke, leukocytes have negative effects on ischemic stroke; some of these effects are poor neurobehavioral outcomes, high infarction volume, and increased blood-brain barrier degradation [2]. Increasing evidence has indicated that CD151 potentially participated in the interaction between endothelial and immune cells [9, 10, 17]. Thus, CD151 was possibly involved in mediating the leukocytic and endothelial interaction to further influence neurological outcomes after the CD151 downregulation. Immunohistochemistry and MPO activity test were conducted to evaluate neutrophil and monocyte infiltration.

**Fig. 2:**
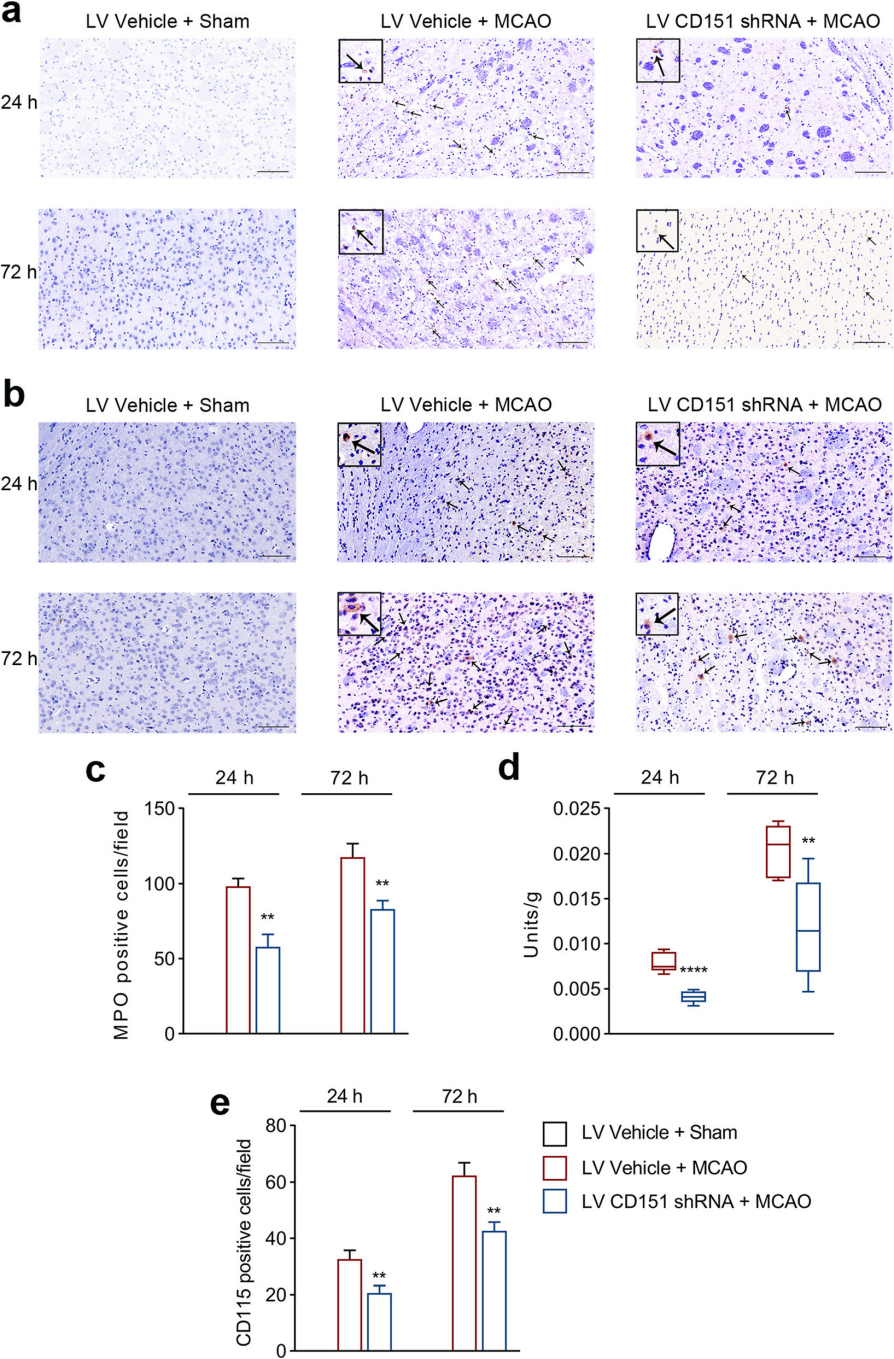
CD151 downregulation alleviated the neutrophil and monocyte infiltration after ischemic stroke. **a** Neutrophil infiltration with a zoomed version (scale bar = 50 μm). Black arrows indicated MPO-positive cells. **b** Monocyte infiltration with a zoomed version (scale bar = 50 μm). Black arrows indicated CD115-positive cells. **c** Neutrophil infiltration decreased 24 and 72 h after reperfusion in vivo (*n* = 3 per group). ** vs. LV Vehicle + MCAO indicates *p* < 0.01. **d** The MPO activity, which was used as a neutrophil activation indicator, was reduced in the LV CD151 shRNA + MCAO group 24 and 72 h after reperfusion in vivo (*n* = 6 per group). ** and **** vs. LV Vehicle + MCAO indicate *p* < 0.01 and 0.0001, respectively. **e** Monocyte infiltration decreased 24 and 72 h after reperfusion in vivo (*n* = 3 per group). ** vs. LV Vehicle + MCAO indicates *p* < 0.01

MPO was used as a marker to identify infiltrating neutrophils in the penumbra [18]. In the sham group, neutrophils were hardly found in the parenchyma. Ischemia led to a significantly increased infiltration of neutrophils, and this phenomenon was reversed by the LV CD151 shRNA administration (Fig. 2a, c). Consistently, the MPO activity test [19] revealed a decreased activity in the LV CD151 shRNA + MCAO group (Fig. 2d). Simultaneously, CD115 was used as a marker to identify the infiltrating monocytes in the penumbra [20]. Monocyte infiltration was largely inhibited by CD151 downregulation compared with that in the LV Vehicle + MCAO group (Fig. 2b, e). Overall, CD151 downregulation distinctly alleviated neutrophil and monocyte infiltration and possibly explained the protective role of the CD151 downregulation in vivo.

### CD151 downregulation influenced VCAM-1 in the endothelial docking structure

Neutrophil infiltration includes several steps, namely, tethering, rolling, adhesion, crawling, and transmigration [21]. Monocyte infiltration involves capture, rolling, slow rolling, arrest, adhesion strengthening, and lateral locomotion [22]. In acute inflammation, the adhesion molecules expressed by endothelial cells (i.e., ICAM-1, VCAM-1, and E-selectin) and their ligands on neutrophils or monocytes mediate the interaction of endothelial cells and neutrophils [21, 22]. However, before adhesion molecules’ ligands are fully presented by neutrophils or monocytes, ICAM-1 and VCAM-1 are already recruited into the endothelial docking structure [10]. This early response is approached by tetraspanin, including CD151 [10]. CD151 forms TEMs, together with CD9, VCAM-1, and ICAM-1, for leukocyte adhesion during inflammation [9].

**Fig. 3:**
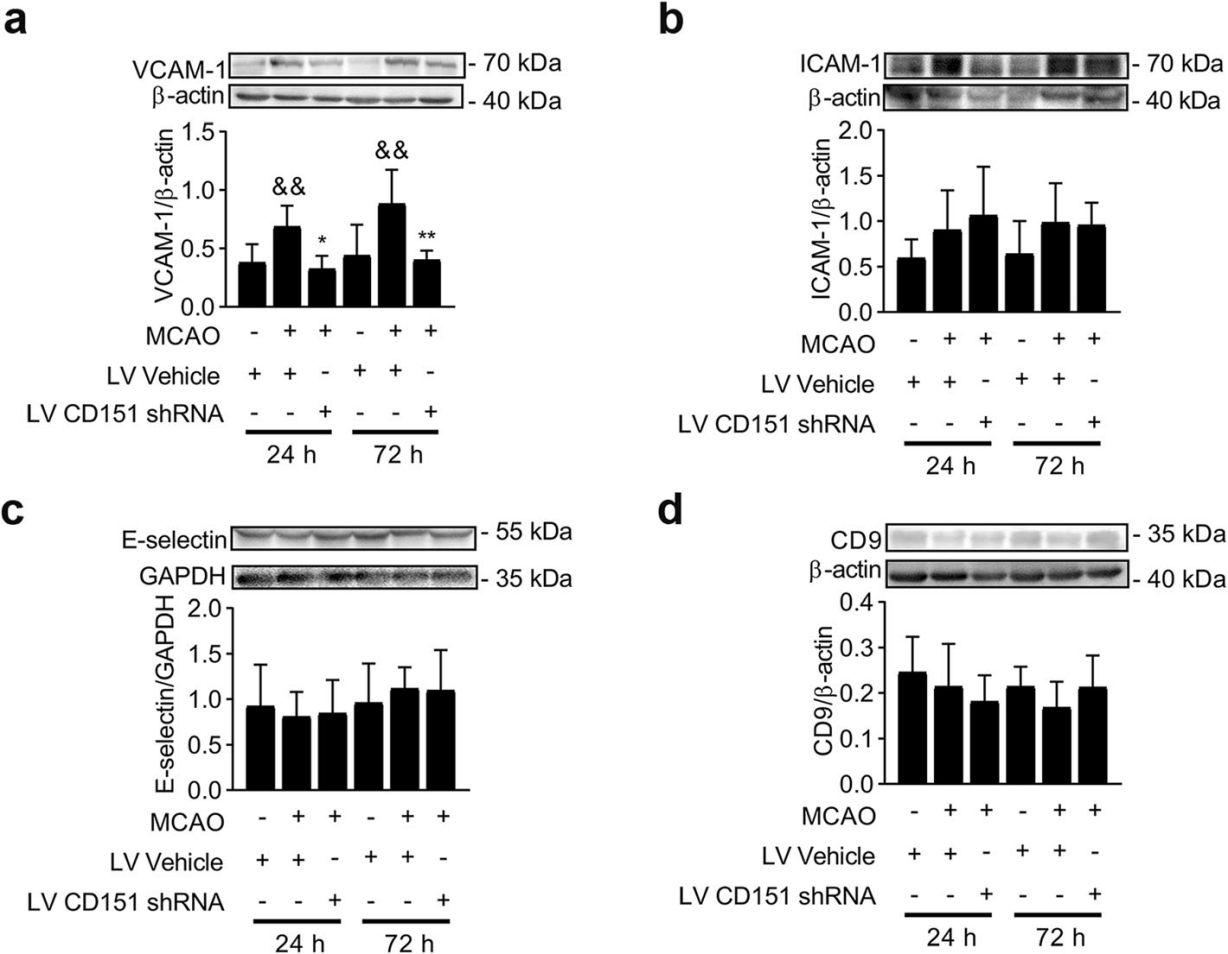
CD151 downregulation influenced the VCAM-1 expression in vivo. **a** VCAM-1, **b** ICAM-1, **c** E-selectin, and **d** CD9 expression levels evaluated using WB in vivo (*n* = 6 per group). * and ** vs. LV Vehicle + MCAO indicates *p* < 0.05 and *p* < 0.01, respectively. & vs. LV Vehicle + sham indicates *p* < 0.05

**Fig. 4:**
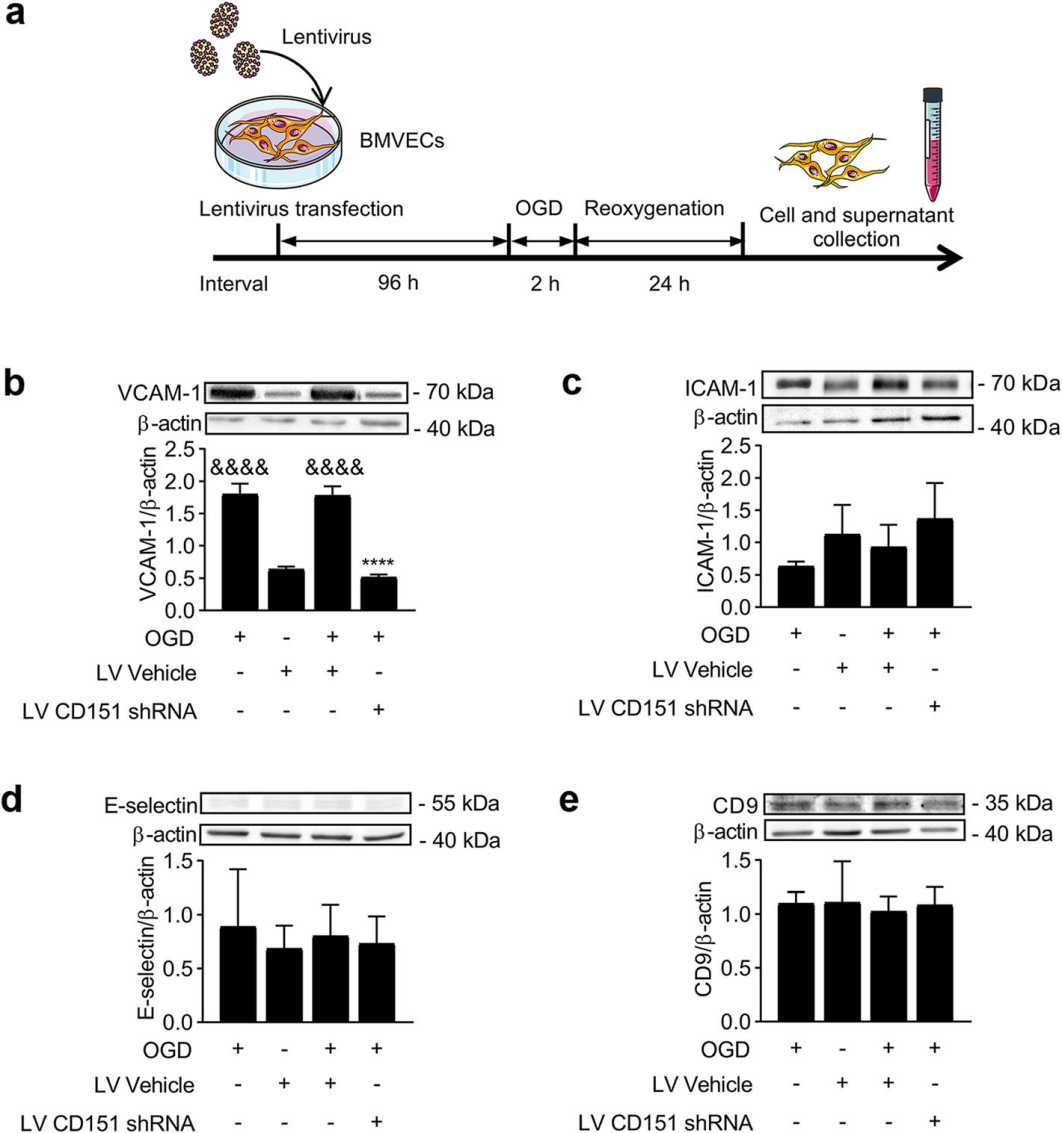
CD151 downregulation influenced the VCAM-1 expression in vitro. **a** In vitro experiment design. BMVECs underwent 2 h OGD and 24 h reoxygenation and collected for protein extraction and tests. **b** VCAM-1, **c** ICAM-1, **d** E- selectin, and **e** CD9 expression levels evaluated using WB in vitro (*n* = 3 per group). **** vs. LV Vehicle + OGD indicates *p* < 0.0001. &&&& vs. LV vehicle indicates *p* < 0.0001

Here, we examined the expression levels of VCAM-1, ICAM-1, E-selectin, and CD9 in vivo and in vitro to confirm the effects of CD151 on endothelial cell adhesion molecules within the TEMs in cerebral ischemia. Consistent with previous reports [23], our study showed that VCAM-1 increased after ischemia both in vivo and in vitro (Figs. 3a and 4a). Interestingly, VCAM-1 significantly decreased, whereas ICAM-1, E-selectin, or CD9 had no significant change in the LV CD151 shRNA + MCAO group (Fig. 3). Overall, the in vitro experiment design is shown in Fig. 4a. In vitro experiments exhibited similar results (Fig. 4b–e). This finding was consistent with previous reports that CD151 preferentially forms a heterodimer with VCAM-1 within TEMs, indicating a close functional relationship between CD151 and VCAM-1 [17].

### Activation of the p38 and NF-κB pathways was inhibited after the CD151 downregulation in endothelial cells

Our data suggested a potential relationship between CD151 and VCAM-1, but the underlying mechanisms of the interaction remained unclear. Studies have shown that mitogen-activated protein kinase (MAPK) and NF-κB pathways are required for VCAM-1 regulation [24]. Thus, the canonical NF-κB pathway exacerbates inflammation and can be activated by MAPK [25]. MAPK kinases, such as p38, JNK, and ERK, function as upstream regulators of NF-κB, and the phosphorylation of MAPK kinases leads to the activation of the canonical NF-κB pathway (IκB α degradation) followed by the NF-κB subunit (p65) translocation from the cytoplasm to the nucleus to promote downstream transcription [26, 27].

**Fig. 5:**
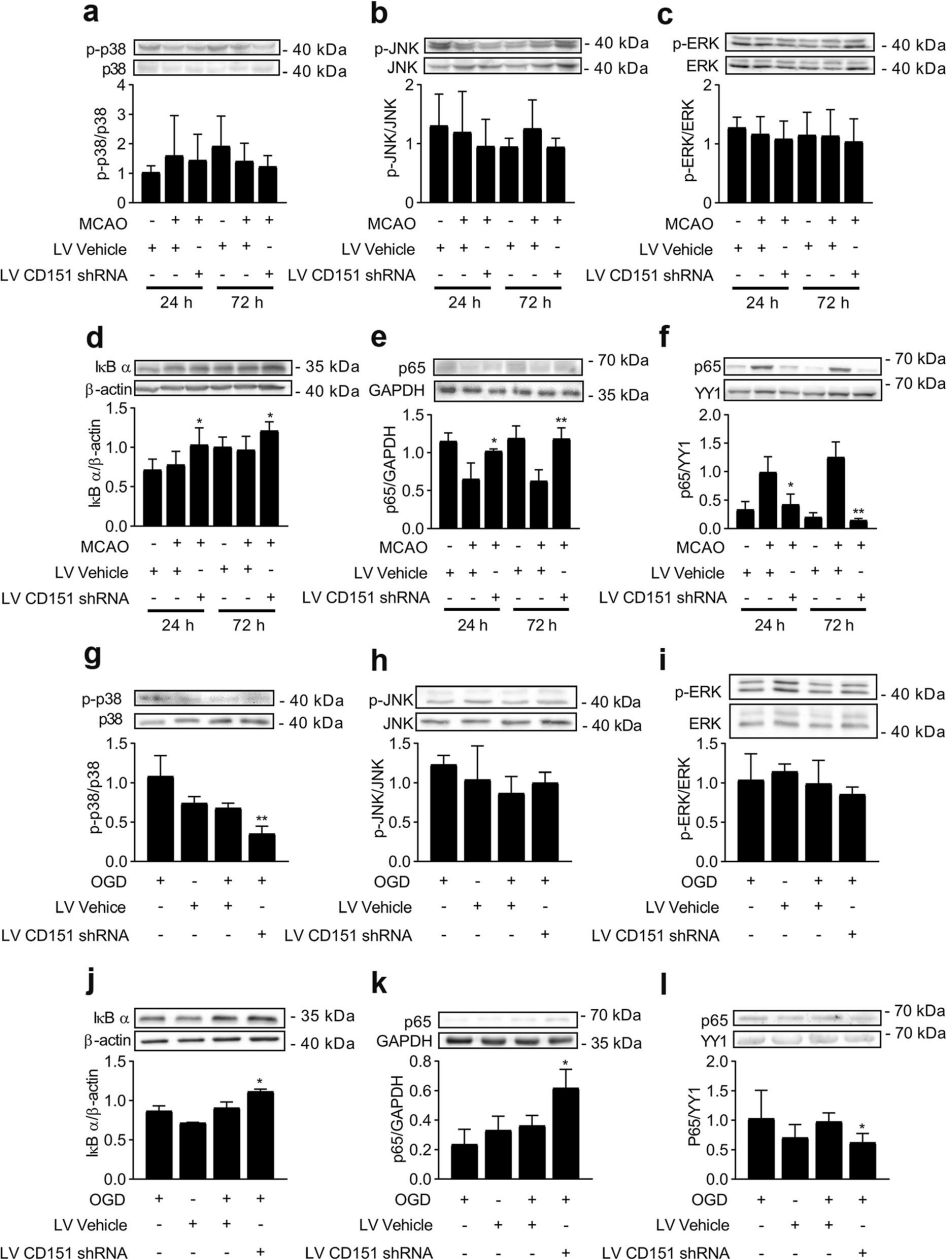
The MAPK and the NF-κB pathways were inhibited after the CD151 downregulation in endothelial cells. The MAPK kinase (i.e., p38, JNK, and ERK) activation was evaluated using **a**–**c** brain tissue (*n* = 6 per group) and **g**–**i** in vitro (*n* = 3 per group) experiments. ** vs. LV Vehicle + MCAO indicates *p* < 0.01. The NF-κB pathway activation evaluated using the **d**, **j** IκB α degeneration and the p65 translocation from the **e**, **k** cytoplasm to the **f**, **l** nucleus in enriched endothelial cells (*n* = 3 per group) and in vitro (*n* = 3 per group). * and ** vs. LV Vehicle + MCAO indicate *p* < 0.05 and 0.01, respectively

In our study, the activation of MAPK kinases (i.e., p38, JNK, and ERK) and NF-κB (i.e., IκB α and P65) was first tested in the infarcted hemisphere in vivo. In vivo data (Fig. 5a–f) revealed that the CD151 downregulation evidently decreased IκB α degradation and p65 nucleus translocation without significantly influencing the MAPK pathways. This result indicated that the activation of the NF-κB pathways was restrained by the CD151 downregulation. However, the reduced activation in the CD151 downregulation group could result from a generally less severe ischemic injury. Furthermore, the influence of the activation of the MAPK and NF-κB pathways within other cells, such as neurons or astrocytes, could not be eliminated. In vitro experiments were designed using cultured primary BMVECs to test the activation of the MAPK and NF-κB pathways (Fig. 5g–l). Notably, the activation of the p38 and NF-κB pathways was restricted. Therefore, the MAPK and NF-κB pathways were involved in the consistent expression of CD151 and VCAM-1 in endothelial cells after cerebral ischemia.

### CD151 downregulation influenced the VCAM-1 expression through the p38/NF-κB pathways in endothelial cells

**Fig. 6:**
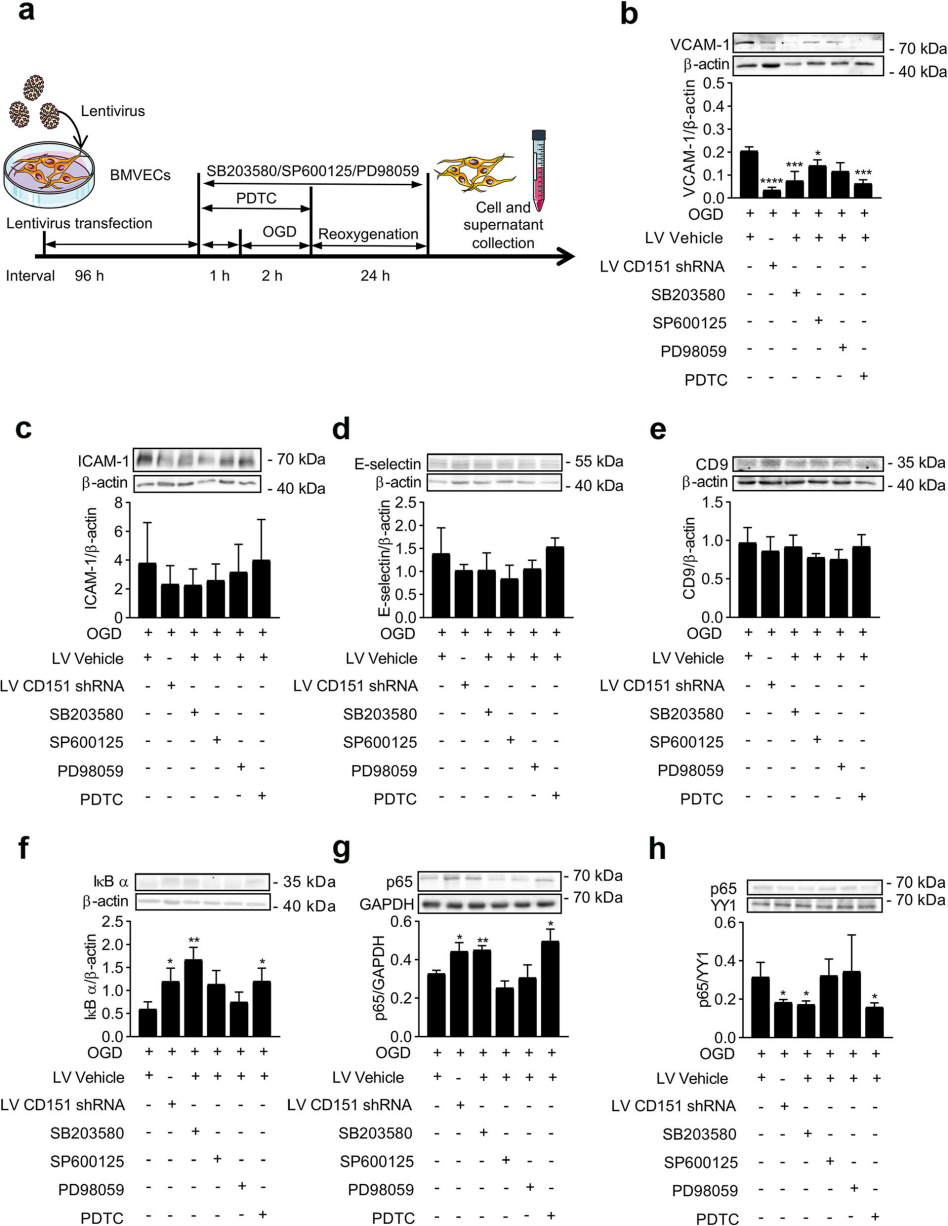
CD151 downregulation influenced the VCAM-1 expression through the p38/NF-κB pathway. **a** In vitro experiment design with inhibitor treatment. SB203580 (p38 inhibitor, 30 μM), SP600125 (JNK inhibitor, 30 μM), PD98059 (ERK 1/2 inhibitor, 30 μM), and PDTC (NF-κB inhibitor, 50 μM) were applied in the LV Vehicle + OGD group. **b** VCAM-1, **c** ICAM-1, **d** E-selectin, and **e** CD9 expression levels after inhibitor application (*n* = 3 per group). *, ***, and **** vs. LV Vehicle + OGD indicate *p* < 0.05, 0.001, and 0.0001, respectively. IκB α degeneration (**f**) and p65 translocation from the **g** cytoplasm to the **h** nucleus were used to evaluate the NF-κB activation (*n* = 3 per group). * and ** vs. LV Vehicle + OGD indicate *p* < 0.05 and 0.01, respectively

After the activation of p38 and the NF-κB pathways were tested to be restricted in the LV CD151 shRNA administration groups, one major concern was the connection between CD151 and the two pathways. Ideally, the restricted p38 and NF-κB pathway activation caused by CD151 downregulation should be rescued by selective agonists. Notably, the selectivity of the reported p38 pathways agonists was not optimal enough, and NF-κB pathway selective agonists were not widely documented. For example, anisomycin was reported as the agonist of both p38 and JNK [28]. Thus, instead of using agonists for rescue experiment designs, in vitro experiments with widely accepted selective antagonists were used for indirect proof as previously reported to explore CD151’s connection with the p38 and NF-κB pathways [26]. p38, JNK, ERK, and NF-κB inhibitors (i.e., SB203580, SP600125, PD98059, and PDTC) were applied to further establish the relationship of CD151, p38, NF-κB, and VCAM-1. BMVECs were pretreated with the aforementioned inhibitors for 1 h and subjected to OGD for 2 h. All the inhibitors except PDTC were added to the BMVEC culture medium during 24 h of reoxygenation for cell endurance consideration (Fig. 6a). As shown in Fig. 6b–e, the p38 or NF-κB inhibition led to a decreased VCAM-1 expression level without significantly influencing on ICAM-1, E-selectin, or CD9 expression level. This result indicated that p38 and NF-κB were at least partially responsible for the change in the VCAM-1 expression. JNK inhibitor itself could also reduce the VCAM-1 expression. In fact, p38, JNK, and ERK are possible upstream regulators of VCAM-1 [24, 29, 30]. However, the decreased JNK activation was not observed in vivo and in vitro after ischemia in the CD151 downregulation groups; as such, JNK was unlikely involved in the CD151’s regulation of VCAM-1. Moreover, the p38 inhibitor inhibited the p65 translocation, suggesting that p38 acted as an upstream regulator of the NF-κB pathway (Fig. 6f–h). Therefore, the CD151 downregulation possibly reduced VCAM-1 at least partially via the p38/NF-κB pathway.

### CD151 downregulation in BMVECs had no direct effect on the activation of neutrophils and monocytes

**Fig. 7:**
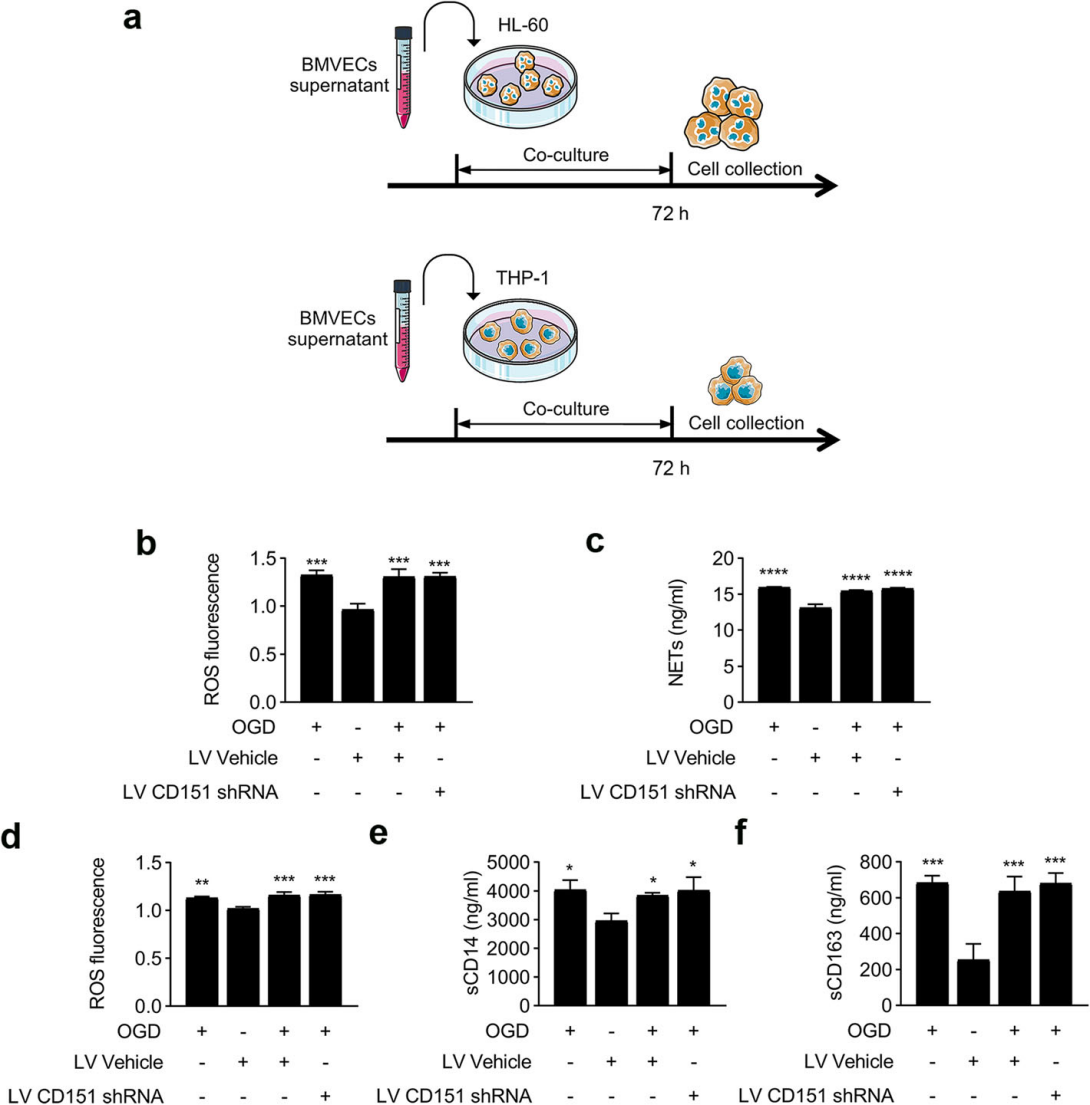
No direct effect was detected in the activation of neutrophil and monocyte after stimulation by the BMVEC supernatant. **a**, **b** In vitro experiment design: the collected BMVEC supernatant was added into the HL-60 cell line or THP-1 culture media and cultured for 72 h. **b** ROS production in HL-60 was tested. *** vs. LV Vehicle + OGD indicate *p* < 0.001. **c** NET formation in HL-60 was detected by ELISA. **** vs. LV Vehicle + OGD indicate *p* < 0.0001. **d** ROS production in THP-1 was tested. ** and *** vs. LV Vehicle + OGD indicate *p* < 0.01 and 0.001, respectively. **e** sCD14 secretion in THP-1 supernatant was detected by ELISA. * vs. LV Vehicle + OGD indicate *p* < 0.05. **f** sCD163 secretion in THP-1 supernatant was detected by ELISA. *** vs. LV Vehicle + OGD indicate *p* < 0.001

Our data revealed the influences of the CD151 downregulation on endothelial cell docking structure during the interaction between endothelial cells and neutrophils. However, no evidence was observed to elucidate the direct effect on neutrophils and monocytes. An in vitro model was utilized to test the activation of neutrophils and monocytes (Fig. 7a). An ROS assay was applied to examine the activation of neutrophils and monocytes. Simultaneously, the NET formation was verified as another activation marker of neutrophil, and sCD14 and Scd163 were considered two soluble markers of monocyte activation [31]. As shown in Fig. 7b–e, the supernatant from all the OGD groups could activate neutrophils and monocytes, and no significant difference was observed in the OGD, LV Vehicle + OGD, and LV CD151 shRNA + OGD. In summary, the influences of CD151 downregulation were likely restricted in BMVECs and might have no direct effect on the activation of neutrophils and monocytes.

## Discussion

In this study, we first demonstrated that the downregulation of CD151 decreased the VCAM-1 on endothelial cells, thereby restraining neutrophil and monocyte infiltration and improving neurological outcomes after cerebral ischemia. Further results suggested that the p38/NF-κB pathways played a major part in the decreased expression of VCAM-1 caused by the CD151 downregulation. Additionally, the CD151 downregulation had no direct effect on neutrophil and monocyte activation, indicating that neutrophils and monocytes might still have normal function. To our knowledge, our work first introduced the therapeutic value of the CD151 downregulation on the interaction between endothelial cells and neutrophils after cerebral ischemia. Our work also provided further evidence on improving the development of current anti-adhesion molecule therapies.

In terms of the translational failure of anti-adhesion molecule therapies, possible explanations have focused on the interferences of immune systems caused by dosage, application time point, or nonhumanized origin of antibodies against adhesion molecule therapy [5, 32]. Although tetraspanin especially CD151 is essential in terms of adhesion molecules’ normal expression and function during leukocyte and endothelial cell interaction, the evaluation of its potential value in improving current adhesion molecule-targeting therapies has yet to be fully explored [9]. Here, we suggested that the VCAM-1 expression and function were also influenced by CD151 through the p38/NF-κB pathway. The p38 MAPK and the NF-κB pathways were involved in neuroinflammation and showed neuronal protection with reduced activation after cerebral ischemia [33, 34]. Thus, the inhibition of both pathways by the CD151 downregulation indicated a limited endothelial cell inflammation, which implied that the CD151 downregulation affected neutrophil and monocyte infiltration and decreased inflammatory responses in endothelial cells. In fact, VCAM-1-targeting therapies are likely effective in reducing ischemia injury in some works, whereas VCAM-1 antibodies show no protective effect in other studies. This observation implies the significance of comprehensive evaluation in adhesion molecule regulation and the importance of the underlying signaling [35–37]. Apparently, our data revealed a complete downstream influence caused by the CD151 downregulation, which could partially explain the contradictory results in VCAM-1-targeting therapies. They might add new evidence to the improvement of current adhesion molecule-targeting therapies. This study also proposed a new therapeutic strategy.

The lack of evidence in the appropriate therapeutic time window of neutrophil-targeting drugs could also explain the translational failures. Previously, leukocyte accumulation after cerebral ischemia was treated as an event initiated after reperfusion [38]. However, intravital imaging data suggested that leukocyte margination is observed as early as 20 to 30 min after ischemia [39], indicating that the leukocyte accumulation can be considered as a result of ischemia rather than reperfusion. Moreover, the risk factors related to cerebral vascular diseases, such as hyperglycemia or high blood pressure, have an intertwined relationship with leukocytes, thereby increasing the neutrophil or monocyte activation or counts even at baseline [40–45]. Thus, focusing on interrupting the endothelial cell and leucocyte interaction at an early stage may prevent severe neurological injuries in patients with underlying diseases, and this phenomenon is frequently observed in clinical situations. Overall, the early application of the medications that target this interaction might be necessary to improve the prognosis of high-risk populations of stroke, such as patients with higher-than-optimum blood glucose or high blood pressure. Therefore, in contrast to previous clinical trials that applied leukocyte infiltration-targeting medication after ischemia [6], our work suggested that the downregulation of CD151 at baseline had a significant effect on hampering neutrophil and monocyte infiltration. Therefore, it showed potential for therapies targeting the interaction between endothelial cells and leukocytes to prevent unfavorable ischemic stroke outcomes.

Another obstacle in leukocyte-targeting therapies was that the complex adhesion molecule interactions between endothelial cells and leukocytes led to reverse results. For example, studies have shown that the application of ICAM-1 antibodies after ischemia leads to an unexpected upregulation of other adhesion molecules and results in a poor local tissue injury [46]. Inversely, our work provided new evidence that the downregulation of CD151 preferentially decreased the VCAM-1 expression without remarkable upregulation on other molecules within TEMs, indicating that CD151 organized the function of TEMs in a relatively controllable way. Moreover, the improper function of TEMs caused by the CD151 downregulation might not directly affect the activation of neutrophils and monocytes, possibly ensuring the neutrophils’ normal function against infection after ischemia. This finding was valuable when we considered the abnormally high rate of pneumonia reported by a previous clinical trial after the usage of ICAM-1 antibody in patients with stroke [47]. Overall, our work presented the influences of the CD151 downregulation preferentially associated with the VCAM-1 reduction and restrained within endothelial cells, which provided a controllable target and diminished potential infective threats because of abnormal neutrophil functions.

Our work had some limitations. The cerebral ischemic model was not fully consistent with patients with ischemic stroke in clinics. Thus, further studies using aged female and male rodents and thromboembolism-induced ischemia model are ongoing. The effect of CD151 in the hyperacute and long-term phase after cerebral ischemia will be examined. Moreover, practical ways, such as the use of CD151 antagonists, will be introduced to regulate CD151 in future studies.

## Conclusion

This work suggested that the CD151 downregulation improved neurological outcomes by restraining neutrophil and monocyte infiltration through the inhibition of the VCAM-1 expression via the p38/NF-κB pathways. It also emphasized the importance of tetraspanin CD151 in regulating adhesion molecules within TEMs in endothelial cells after experimental stroke. This therapeutic strategy might show potential for the development of anti-adhesion molecule therapies to control leukocyte infiltration in ischemic stroke.

## Supplementary Information

**Additional file 1: Supplementary method.** RNA extraction and reverse transcriptase-polymerase chain reaction (RT-PCR). **Table S1.** Detailed information of lentivirus CD151 shRNA sequences. **Figure S1.** Different lentivirus CD151 shRNA transfection effectiveness *in vitro*. **Figure S2.** Lentivirus CD151 shRNA transfection effectiveness assessment *in vivo*. **Figure S3.** Lentivirus transfection effectiveness *in vitro*. **Figure S4.** CD151 expression assessment at observation time points *in vivo* and *in vitro.* **Figure S5.** p38 and NF-κB activation were restrained *in vivo* after CD151 knockdown. The MAPK kinase (i.e., p38, JNK, and ERK) activation was evaluated (a, b, c) using infarcted hemisphere (*n* = 6 per group) or enriched endothelial cells (g, h) from infarcted hemisphere (*n* = 3 per group), * and **vs. LV Vehicle + MCAO indicate *p* < 0.05 and 0.01, respectively. The NF-κB pathway activation evaluated using the (d) IκB α degeneration and the p65 translocation from the (e) cytoplasm to the (f) nucleus in infarcted hemisphere (n = 6 per group), *, ** and *** vs. LV Vehicle + MCAO indicate p < 0.05, 0.01 and 0.001, respectively. **Figure S6.** Anisomycin increased the phosphorylation of both p38 and JNK in BMVECs. Cultured primary BMVECs were treated with 1μM anisomycin for 3 h (n = 3 per group). An increase in the phosphorylation of p38 (a) and JNK (b) were observed, ** vs. control group indicate p < 0.01.

## Data Availability

The datasets used and/or analyzed during the current study are available from the corresponding author on reasonable request.

## Abbreviations

BMVEC: Primary rats brain microvascular endothelial cells
BSA: Bovine serum albumin
DAB: Diaminobenzidine
ELISA: Enzyme-linked immunosorbent assay
ERK: Extracellular-related protein kinases
GAPDH: Glyceraldehyde-3-phosphate dehydrogenase
GFP: Green fluorescent protein
ICAM-1: Intercellular cell adhesion molecule-1
ICV: Intrasubventricular injection
IκB α: NF-κB inhibitor α
JNK: Jun N-terminal kinase
MAPK: Mitogen-activated protein kinase
MCAO: Middle cerebral artery occlusion
MPO: Myeloperoxidase
NF-κB: Nuclear factor kappa-B
NETs: Neutrophil extracellular traps
OGD: Oxygen glucose deprivation
p38: p38 mitogen-activated protein kinase
PBS: Phosphate-buffered saline
PFA: Paraformaldehyde
ROS: Reactive oxygen species
SD: Sprague-Dawley
SPF: Specific pathogen-free
TEMs: Tetraspanin-enriched domain
TTC: 2,3,5-Triphenyltetrazolium chloride
TUNEL: Terminal deoxynucleotidyl transferase dUTP nick end labeling
VCAM-1: Vascular cell adhesion molecule-1

## References

[1] Jayaraj RL, Azimullah S, Beiram R, Jalal FY, Rosenberg GA (2019). Neuroinflammation: friend and foe for ischemic stroke. J Neuroinflammation..

[2] Jickling GC, Liu D, Ander BP, Stamova B, Zhan X, Sharp FR (2015). Targeting neutrophils in ischemic stroke: translational insights from experimental studies. J Cereb Blood Flow Metab..

[3] Liberale L, Montecucco F, Bonaventura A, Casetta I, Seraceni S, Trentini A, Padroni M, Dallegri F, Fainardi E, Carbone F (2017). Monocyte count at onset predicts poststroke outcomes during a 90-day follow-up. Eur J Clin Invest..

[4] Grønberg NV, Johansen FF, Kristiansen U, Hasseldam H (2013). Leukocyte infiltration in experimental stroke. J Neuroinflammation..

[5] Frijns CJ, Kappelle LJ (2002). Inflammatory cell adhesion molecules in ischemic cerebrovascular disease. Stroke..

[6] Veltkamp R, Gill D (2016). Clinical trials of immunomodulation in ischemic stroke. Neurotherapeutics..

[7] Wadkin J, Patten DA, Kamarajah SK (2017). CD151 supports VCAM-1-mediated lymphocyte adhesion to liver endothelium and is upregulated in chronic liver disease and hepatocellular carcinoma. Am J Physiol Gastrointest Liver Physiol..

[8] Hemler ME (2003). Tetraspanin proteins mediate cellular penetration, invasion, and fusion events and define a novel type of membrane microdomain. Annu Rev Cell Dev Biol..

[9] Barreiro O, Yáñez-Mó M, Sala-Valdés M (2005). Endothelial tetraspanin microdomains regulate leukocyte firm adhesion during extravasation. Blood..

[10] Ley K, Zhang H (2008). Dances with leukocytes: how tetraspanin-enriched microdomains assemble to form endothelial adhesive platforms. J Cell Biol..

[11] Longa EZ, Weinstein PR, Carlson S, Cummins R (1989). Reversible middle cerebral artery occlusion without craniectomy in rats. Stroke..

[12] Kramer M, Dang J, Baertling F, Denecke B, Clarner T, Kirsch C, Beyer C, Kipp M (2010). TTC staining of damaged brain areas after MCA occlusion in the rat does not constrict quantitative gene and protein analyses. J Neurosci Methods..

[13] Li M, Wen Y, Zhang R, Xie F, Zhang G, Qin X (2018). Adenoviral vector-induced silencing of RGMa attenuates blood-brain barrier dysfunction in a rat model of MCAO/reperfusion. Brain Res Bull..

[14] Wu Y, Wang L, Dai C, Ma G, Zhang Y, Zhang X, Wu Z (2014). Neuroprotection by platelet-activating factor acetylhydrolase in a mouse model of transient cerebral ischemia. Neurosci Lett..

[15] Jolkkonen J, Puurunen K, Rantakömi S, Härkönen A, Haapalinna A, Sivenius J (2000). Behavioral effects of the alpha(2)-adrenoceptor antagonist, atipamezole, after focal cerebral ischemia in rats. Eur J Pharmacol..

[16] Ran Y, Liu Z, Huang S, Shen J, Li F, Zhang W, Chen C, Geng X, Ji Z, du H, Hu X (2018). Splenectomy fails to provide long-term protection against ischemic stroke. Aging Dis..

[17] Barreiro O, Zamai M, Yáñez-Mó M (2008). Endothelial adhesion receptors are recruited to adherent leukocytes by inclusion in preformed tetraspanin nanoplatforms. J Cell Biol..

[18] Zhou W, Liesz A, Bauer H, Sommer C, Lahrmann B, Valous N, Grabe N, Veltkamp R (2013). Postischemic brain infiltration of leukocyte subpopulations differs among murine permanent and transient focal cerebral ischemia models. Brain Pathol..

[19] Weston RM, Jarrott B, Ishizuka Y, Callaway JK (2006). AM-36 modulates the neutrophil inflammatory response and reduces breakdown of the blood brain barrier after endothelin-1 induced focal brain ischaemia. Br J Pharmacol..

[20] Martinez FO, Combes TW, Orsenigo F, Gordon S (2020). Monocyte activation in systemic Covid-19 infection: assay and rationale. EBioMedicine..

[21] Kolaczkowska E, Kubes P (2013). Neutrophil recruitment and function in health and inflammation. Nat Rev Immunol..

[22] Gerhardt T, Ley K (2015). Monocyte trafficking across the vessel wall. Cardiovasc Res..

[23] Wang Y, Su Y, Lai W, Huang X, Chu K, Brown J, Hong G (2020). Salidroside restores an anti-inflammatory endothelial phenotype by selectively inhibiting endothelial complement after oxidative stress. Inflammation..

[24] Lee BK, Lee WJ, Jung YS. Chrysin attenuates VCAM-1 expression and monocyte adhesion in lipopolysaccharide-stimulated brain endothelial cells by preventing NF-κB signaling. Int J Mol Sci. 2017;18(7):1424. 10.3390/ijms18071424 PMC5535915 28671640

[25] Simmons LJ, Surles-Zeigler MC, Li Y, Ford GD, Newman GD, Ford BD (2016). Regulation of inflammatory responses by neuregulin-1 in brain ischemia and microglial cells in vitro involves the NF-kappa B pathway. J Neuroinflammation..

[26] Qin S, Yang C, Huang W, du S, Mai H, Xiao J, Lü T (2018). Sulforaphane attenuates microglia-mediated neuronal necroptosis through down-regulation of MAPK/NF-κB signaling pathways in LPS-activated BV-2 microglia. Pharmacol Res..

[27] Tao L, Li D, Liu H, Jiang F, Xu Y, Cao Y, Gao R, Chen G (2018). Neuroprotective effects of metformin on traumatic brain injury in rats associated with NF-κB and MAPK signaling pathway. Brain Res Bull..

[28] Nikaido M, Otani T, Kitagawa N, Ogata K, Iida H, Anan H, Inai T (2019). Anisomycin, a JNK and p38 activator, suppresses cell-cell junction formation in 2D cultures of K38 mouse keratinocyte cells and reduces claudin-7 expression, with an increase of paracellular permeability in 3D cultures. Histochem Cell Biol.

[29] Chen WC, Lin CY, Kuo SJ, Liu SC, Lu YC, Chen YL, et al. Resistin enhances VCAM-1 expression and monocyte adhesion in human osteoarthritis synovial fibroblasts by inhibiting MiR-381 expression through the PKC, p38, and JNK signaling pathways. Cells. 2020;9(6):1369. 10.3390/cells9061369. 10.3390/cells9061369 PMC7349127 32492888

[30] Yang H, Li X, Liu Y, Li X, Li X, Wu M, Lv X, Chunhua C, Ding X, Zhang Y (2018). Crocin improves the endothelial function regulated by Kca3.1 through ERK and Akt signaling pathways. Cell Physiol Biochem.

[31] Liang H, Xie Z, Shen T (2017). Monocyte activation and cardiovascular disease in HIV infection. Cell Mol Immunol..

[32] Furuya K, Takeda H, Azhar S, McCarron RM, Chen Y, Ruetzler CA, Wolcott KM, DeGraba TJ, Rothlein R, Hugli TE, del Zoppo GJ, Hallenbeck JM (2001). Examination of several potential mechanisms for the negative outcome in a clinical stroke trial of enlimomab, a murine anti-human intercellular adhesion molecule-1 antibody: a bedside-to-bench study. Stroke..

[33] Barone FC, Irving EA, Ray AM, Lee JC, Kassis S, Kumar S, Badger AM, Legos JJ, Erhardt JA, Ohlstein EH, Hunter AJ, Harrison DC, Philpott K, Smith BR, Adams JL, Parsons AA (2001). Inhibition of p38 mitogen-activated protein kinase provides neuroprotection in cerebral focal ischemia. Med Res Rev..

[34] Harari OA, Liao JK (2010). NF-κB and innate immunity in ischemic stroke. Ann N Y Acad Sci..

[35] Liesz A, Zhou W, Mracskó É, Karcher S, Bauer H, Schwarting S, Sun L, Bruder D, Stegemann S, Cerwenka A, Sommer C, Dalpke AH, Veltkamp R (2011). Inhibition of lymphocyte trafficking shields the brain against deleterious neuroinflammation after stroke. Brain..

[36] Justicia C, Martín A, Rojas S, Gironella M, Cervera Á, Panés J, Chamorro Á, Planas AM (2006). Anti-VCAM-1 antibodies did not protect against ischemic damage either in rats or in mice. J Cereb Blood Flow Metab..

[37] Zera KA, Buckwalter MS (2020). The local and peripheral immune responses to stroke: implications for therapeutic development. Neurotherapeutics..

[38] Nour M, Scalzo F, Liebeskind DS (2013). Ischemia-reperfusion injury in stroke. Interv Neurol..

[39] Desilles JP, Syvannarath V, Di Meglio L, et al. Downstream microvascular thrombosis in cortical venules is an early response to proximal cerebral arterial occlusion. J Am Heart Assoc. 2018;7(5):e007804. 10.1161/JAHA.117.007804 PMC5866327 29496683

[40] Desilles JP, Syvannarath V, Ollivier V, Journé C, Delbosc S, Ducroux C, Boisseau W, Louedec L, di Meglio L, Loyau S, Jandrot-Perrus M, Potier L, Michel JB, Mazighi M, Ho-Tin-Noé B (2017). Exacerbation of thromboinflammation by hyperglycemia precipitates cerebral infarct growth and hemorrhagic transformation. Stroke..

[41] Siedlinski M, Jozefczuk E, Xu X, Teumer A, Evangelou E, Schnabel RB, Welsh P, Maffia P, Erdmann J, Tomaszewski M, Caulfield MJ, Sattar N, Holmes MV, Guzik TJ (2020). White blood cells and blood pressure: a Mendelian randomization study. Circulation..

[42] Flint AC, Conell C, Ren X, Banki NM, Chan SL, Rao VA, Melles RB, Bhatt DL (2019). Effect of systolic and diastolic blood pressure on cardiovascular outcomes. N Engl J Med..

[43] Danaei G, Lawes CM, Vander Hoorn S, Murray CJ, Ezzati M (2006). Global and regional mortality from ischaemic heart disease and stroke attributable to higher-than-optimum blood glucose concentration: comparative risk assessment. Lancet..

[44] Loperena R, Van Beusecum JP, Itani HA, Engel N, Laroumanie F, Xiao L, Elijovich F, Laffer CL, Gnecco JS, Noonan J (2018). Hypertension and increased endothelial mechanical stretch promote monocyte differentiation and activation: roles of STAT3, interleukin 6 and hydrogen peroxide. Cardiovasc Res..

[45] Almubarak A, Tanagala K, Papapanou PN, Lalla E, Momen-Heravi F (2020). Disruption of monocyte and macrophage homeostasis in periodontitis. Front Immunol..

[46] DeGraba TJ (1998). The role of inflammation after acute stroke: utility of pursuing anti-adhesion molecule therapy. Neurology..

[47] Enlimomab Acute Stroke Trial Investigators (2001). Use of anti-ICAM-1 therapy in ischemic stroke: results of the Enlimomab Acute Stroke Trial. Neurology..

