# Supplementary material for: Downregulation of CD151 restricts VCAM-1 mediated leukocyte infiltration to reduce neurobiological injuries after experimental stroke
Source: J Neuroinflammation. 2021 May 22;18:118. doi: 10.1186/s12974-021-02171-6 (PMC8140507; doi:10.1186/s12974-021-02171-6)
Supplement: Supplementary file 1 — Additional file 1: Supplementary method. RNA extraction and reverse transcriptase-polymerase chain reaction (RT-PCR). Table S1. Detailed information of lentivirus CD151 shRNA sequences. Figure S1. Different lentivirus CD151 shRNA transfection effectiveness in vitro. Figure S2. Lentivirus CD151 shRNA transfection effectiveness assessment in vivo. Figure S3. Lentivirus transfection effectiveness in vitro. Figure S4. CD151 expression assessment at observation time points in vivo and in vitro. Figure S5. p38 and NF-κB activation were restrained in vivo after CD151 knockdown. The MAPK kinase (i.e., p38, JNK, and ERK) activation was evaluated (a, b, c) using infarcted hemisphere (n = 6 per group) or enriched endothelial cells (g, h) from infarcted hemisphere (n = 3 per group), * and **vs. LV Vehicle + MCAO indicate p < 0.05 and 0.01, respectively. The NF-κB pathway activation evaluated using the (d) IκB α degeneration and the p65 translocation from the (e) cytoplasm to the (f) nucleus in infarcted hemisphere (n = 6 per group), *, ** and *** vs. LV Vehicle + MCAO indicate p < 0.05, 0.01 and 0.001, respectively. Figure S6. Anisomycin increased the phosphorylation of both p38 and JNK in BMVECs. Cultured primary BMVECs were treated with 1μM anisomycin for 3 h (n = 3 per group). An increase in the phosphorylation of p38 (a) and JNK (b) were observed, ** vs. control group indicate p < 0.01. [file 12974_2021_2171_MOESM1_ESM.zip › Revised Additional file 1.docx]

**Supplementary Materials**

**Downregulation of CD151 restricts VCAM-1 mediated neutrophil infiltration to reduce neurobiological injuries after experimental stroke**

Ceshu Gao, Wangyue Jia, Wendeng Xu, Qiong Wu, Jian Wu

Corresponding Authors: Qiong Wu, School of Life Sciences, Tsinghua University, Life Science New Building, Tsinghua Yuan No. 1, Haidian District, Beijing 100084, China. E-mail: [wuqiong@mail.tsinghua.edu.cn](mailto:wuqiong@mail.tsinghua.edu.cn); Jian Wu, Department of Neurology, Beijing Tsinghua Changgung Hospital, School of Clinical Medicine, Tsinghua University, Beijing, China. Email: wujianthu@mail.tsinghua.edu.cn

**This file contains:**

Supplementary Method

Supplementary Table 1

Supplementary Figures

**Supplementary Method**

**RNA extraction and reverse transcriptase-polymerase chain reaction (RT-PCR)**

Total RNA was isolated using TRNzol reagent (Tiangen biotech., China) followed manufacturer’s standard protocol. The reverse-transcriptase reaction was carried out using StarScript II first-strand cDNA synthesis mix with gDNA remover reagent (GenStar Biosolutions Co., China). The reverse-transcriptase reaction was carried out using M-MLV reverse transcriptase (Invitrogen, USA). Quantitative PCR was performed on a 7500 Fast Real-time PCR system (Applied Biosystems, USA) using two-step RT-PCR program parameters provided by the manufacturer. Each sample was tested in triplicate. The following primers for RT-PCR were designed using primerbank and NCBI. CD151: Fwd: ACTATCAGCAGCTCAACACCG; Rev: AGCTTGTCTACAGCGTTGGT.

**Supplementary Table 1** Detailed information of shRNA sequences

|  | Sequence（5'-3'） |
| --- | --- |
| LV CD151 shRNA 1 | GCCTCAAGTATCTGCTGTT |
| LV CD151 shRNA 2 | GCATCCTGGCCTATGTCTA |
| LV CD151 shRNA 3 | GCACGCCTCCAACATCTAT |
| LV Vehicle | TTCTCCGAACGTGTCACGT |

LV: lentivirus


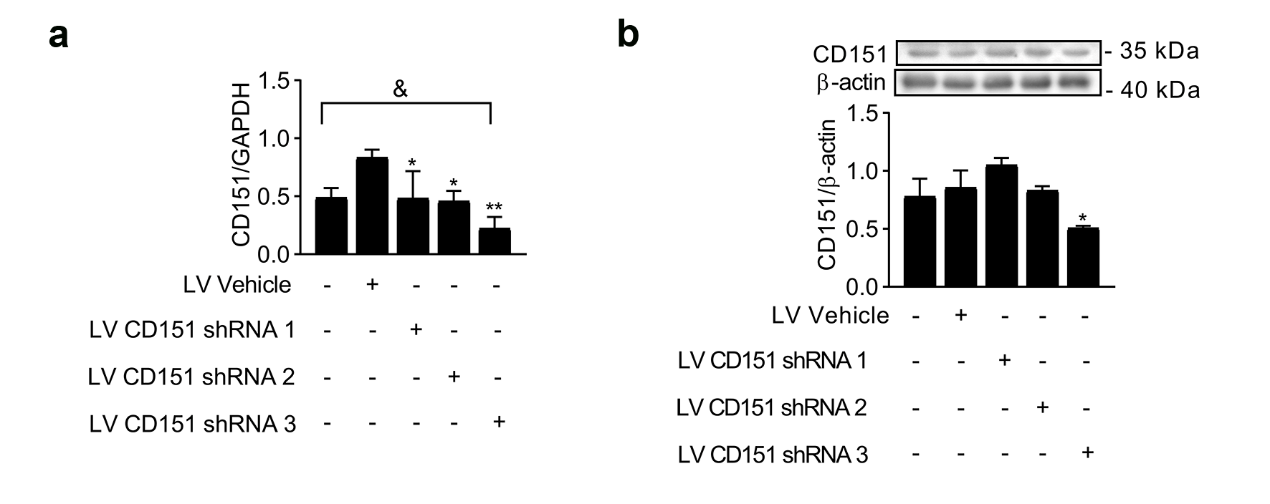


**Supplementary Fig.1** Different lentivirus CD151 shRNA transfection effectiveness *in vitro*. CD151 expression was tested by (a) RT-PCR and (b) WB (*n* = 3 per group), * and ** vs. LV Vehicle indicate *p* < 0.05, 0.01 respectively. & vs. normal cultured cells indicate *p* < 0.05


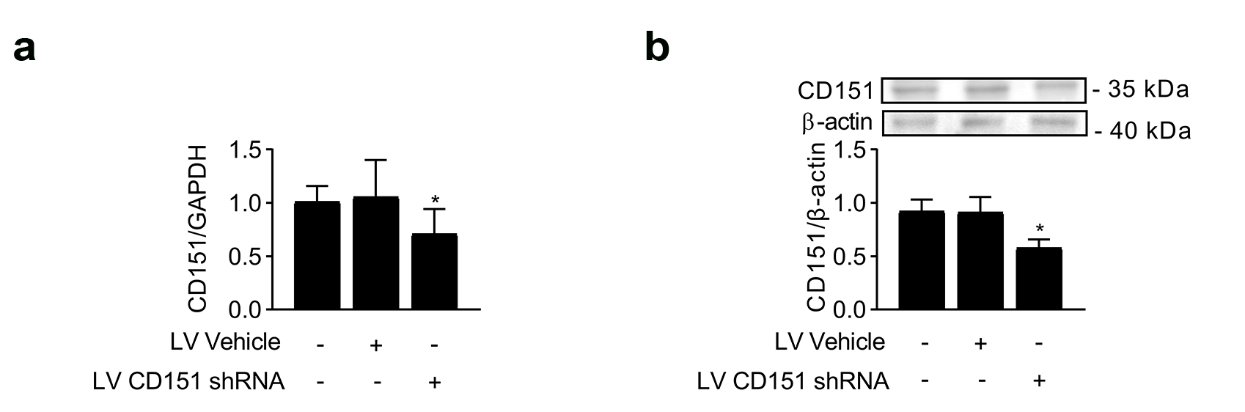


**Supplementary Fig. 2** CD151 expression at seven days after lentivirus subventricular injection *in vivo*. CD151 expression was tested by (a) RT-PCR (*n* = 3 per group) and (b) WB (*n* = 3 per group), * vs. LV Vehicle + MCAO indicate *p* < 0.05

­­


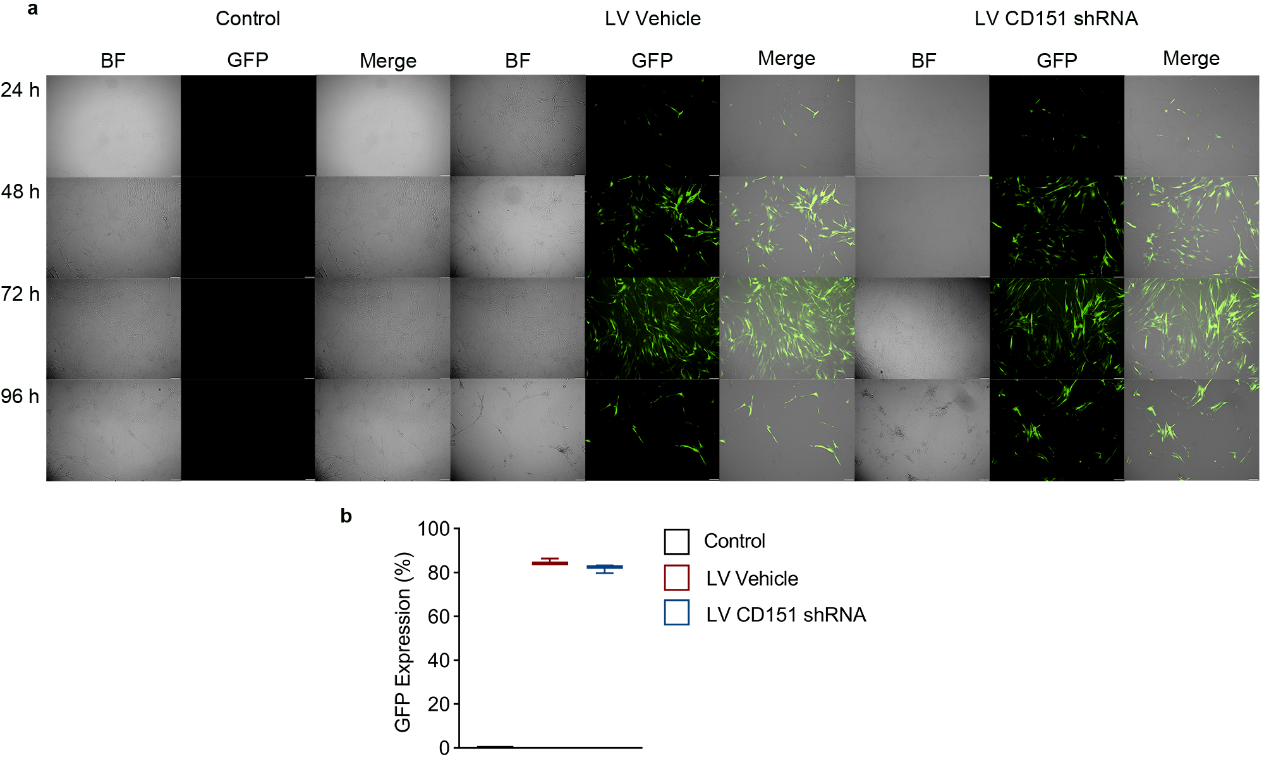


**Supplementary Fig. 3** Lentivirus transfection effectiveness *in vitro*. (a) GFP expression was observed by immunofluorescence (scale bar: 100 μm). (b) No statistically significance of GFP positive cells percentage between LV Vehicle and LV CD151 shRNA was found.


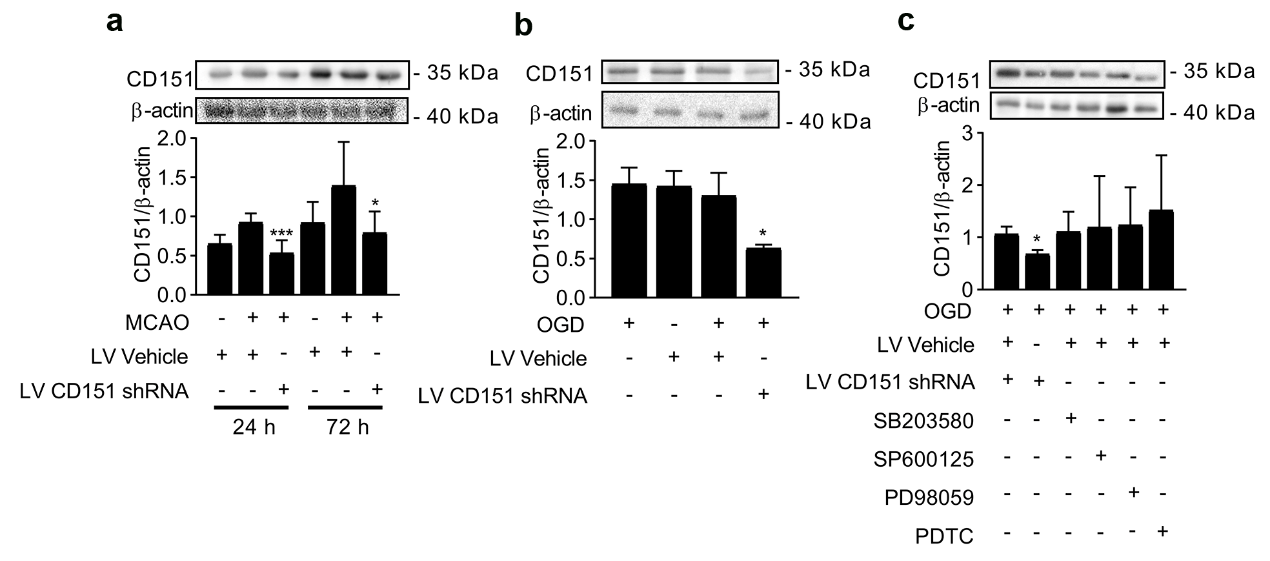


**Supplementary Fig 4** CD151 expression assessment at observation time points *in vivo* and *in vitro*, * and *** vs. LV Vehicle + MCAO or LV Vehicle + OGD indicate *p* < 0.05, 0.001, respectively.


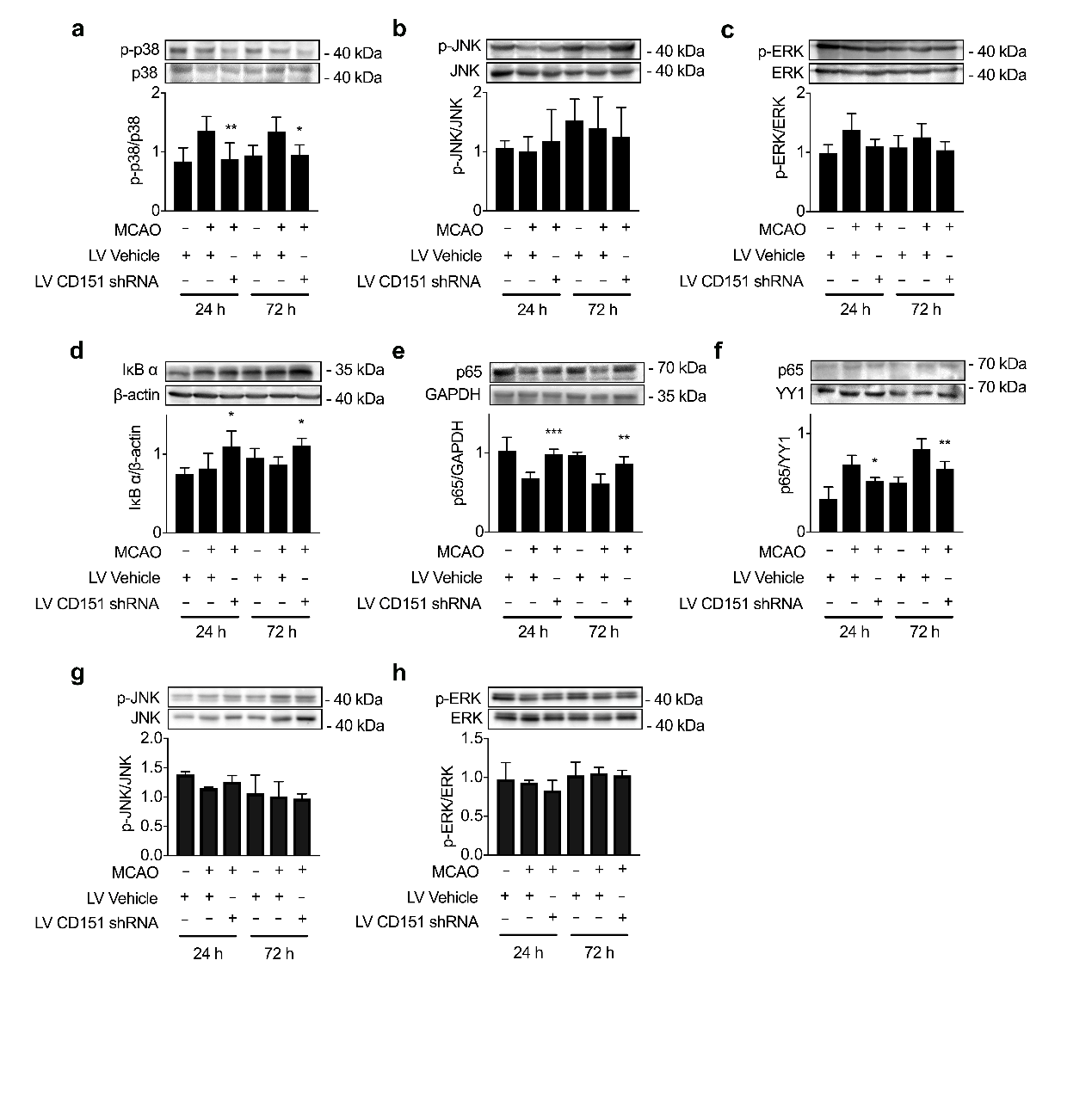


**Supplementary Fig 5** p38 and NF-κB activation were restrained *in vivo* after CD151 knockdown. The MAPK kinase (i.e., p38, JNK, and ERK) activation was evaluated (a, b, c) using infarcted hemisphere (*n* = 6 per group) or enriched endothelial cells (g, h) from infarcted hemisphere (*n* = 3 per group), * and **vs. LV Vehicle + MCAO indicate *p* < 0.05 and 0.01, respectively. The NF-κB pathway activation evaluated using the (d) IκB α degeneration and the p65 translocation from the (e) cytoplasm to the (f) nucleus in infarcted hemisphere (n = 6 per group), *, ** and *** vs. LV Vehicle + MCAO indicate p < 0.05, 0.01 and 0.001, respectively


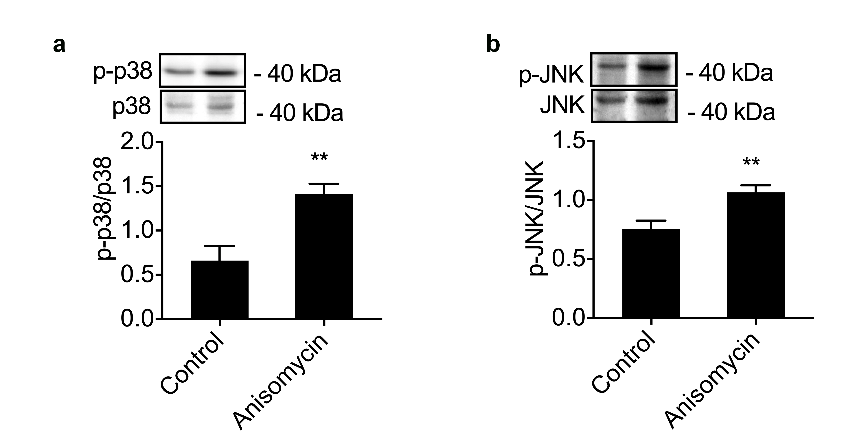


**Supplementary Fig 6** Anisomycin increased the phosphorylation of both p38 and JNK in BMVECs. Cultured primary BMVECs were treated with 1μM anisomycin for 3 h (n = 3 per group). An increase in the phosphorylation of p38 (a) and JNK (b) were observed, ** vs. control group indicate p < 0.01
