# Supplementary material for: Downregulation of CD151 restricts VCAM-1 mediated leukocyte infiltration to reduce neurobiological injuries after experimental stroke
Source: J Neuroinflammation. 2021 May 22;18:118. doi: 10.1186/s12974-021-02171-6 (PMC8140507; doi:10.1186/s12974-021-02171-6)
Supplement: Supplementary file 1 — Additional file 1: Supplementary method. RNA extraction and reverse transcriptase-polymerase chain reaction (RT-PCR). Table S1. Detailed information of lentivirus CD151 shRNA sequences. Figure S1. Different lentivirus CD151 shRNA transfection effectiveness in vitro. Figure S2. Lentivirus CD151 shRNA transfection effectiveness assessment in vivo. Figure S3. Lentivirus transfection effectiveness in vitro. Figure S4. CD151 expression assessment at observation time points in vivo and in vitro. Figure S5. p38 and NF-κB activation were restrained in vivo after CD151 knockdown. The MAPK kinase (i.e., p38, JNK, and ERK) activation was evaluated (a, b, c) using infarcted hemisphere (n = 6 per group) or enriched endothelial cells (g, h) from infarcted hemisphere (n = 3 per group), * and **vs. LV Vehicle + MCAO indicate p < 0.05 and 0.01, respectively. The NF-κB pathway activation evaluated using the (d) IκB α degeneration and the p65 translocation from the (e) cytoplasm to the (f) nucleus in infarcted hemisphere (n = 6 per group), *, ** and *** vs. LV Vehicle + MCAO indicate p < 0.05, 0.01 and 0.001, respectively. Figure S6. Anisomycin increased the phosphorylation of both p38 and JNK in BMVECs. Cultured primary BMVECs were treated with 1μM anisomycin for 3 h (n = 3 per group). An increase in the phosphorylation of p38 (a) and JNK (b) were observed, ** vs. control group indicate p < 0.01. [file 12974_2021_2171_MOESM1_ESM.zip › Western Blot raw data Figure 5.pdf]

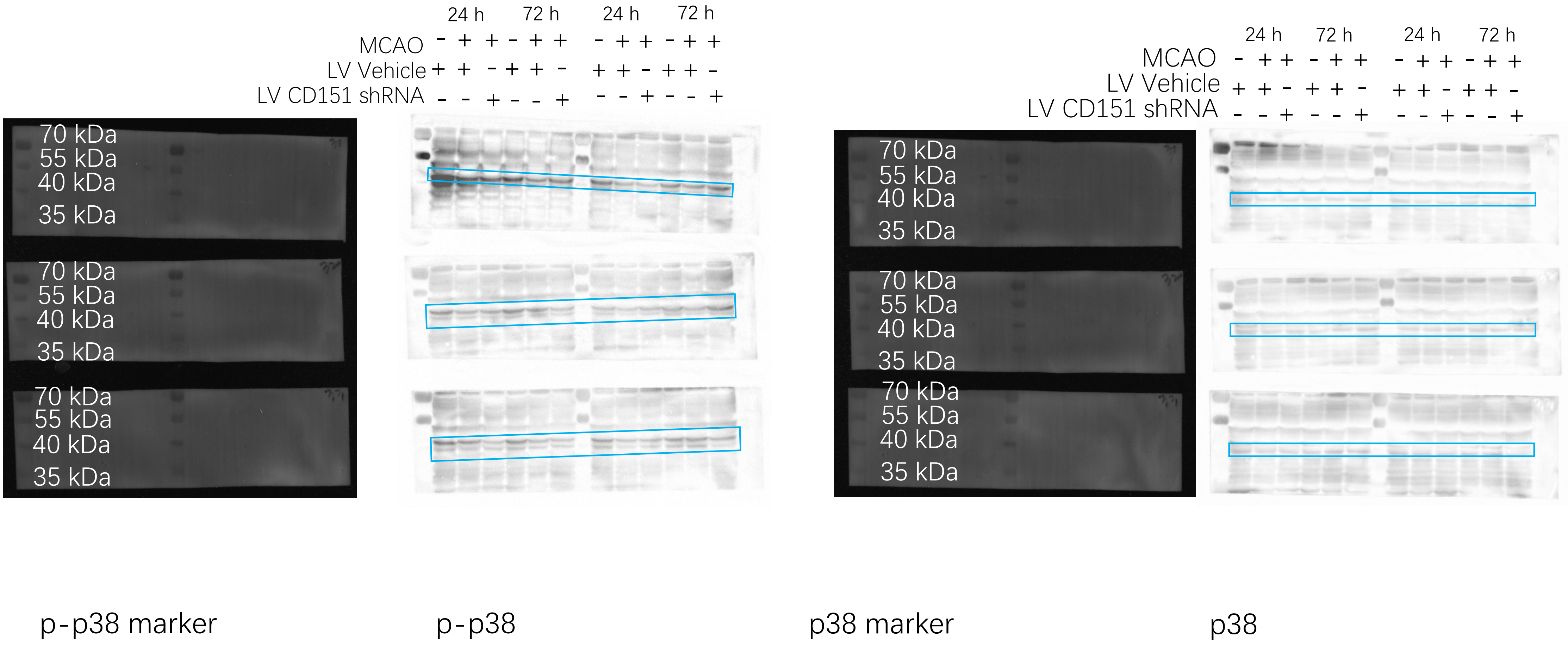
24 h
72 h
24 h
72 h
MCAO - + + - + + - + + - + +
LV Vehicle + + - + + - + + - + + -
LV CD151 shRNA - - + - - + - - + - - +
70 kDa
55 kDa
40 kDa
35 kDa
p-p38 marker
p-p38
p38 marker
p38

Fig 5a

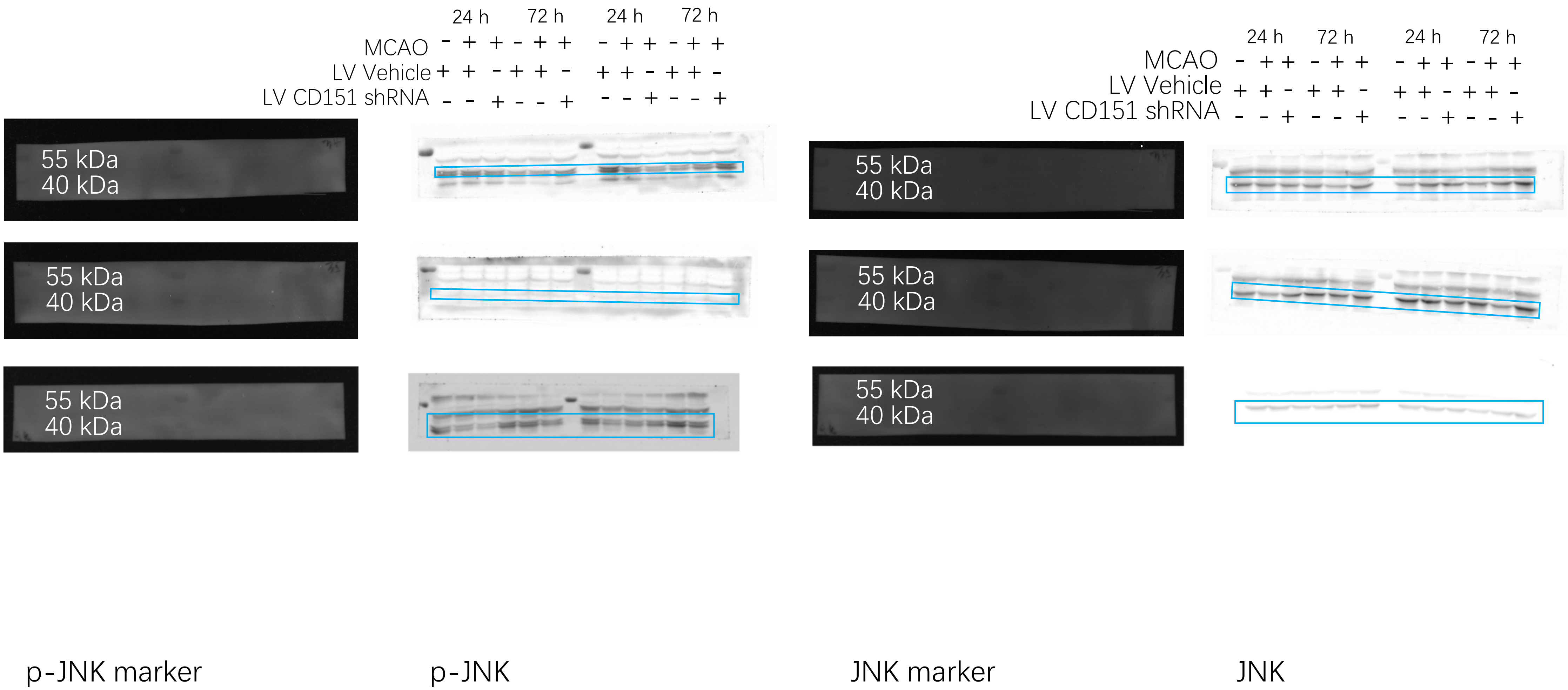
24 h
72 h
24 h
72 h
MCAO - + + - + + - + + - + +
LV Vehicle + + - + + - + + - + + -
LV CD151 shRNA - - + - - + - - + - - +
55 kDa
40 kDa
55 kDa
40 kDa
55 kDa
40 kDa
55 kDa
40 kDa
55 kDa
40 kDa
55 kDa
40 kDa
p-JNK marker
p-JNK
JNK marker
JNK

Fig 5b

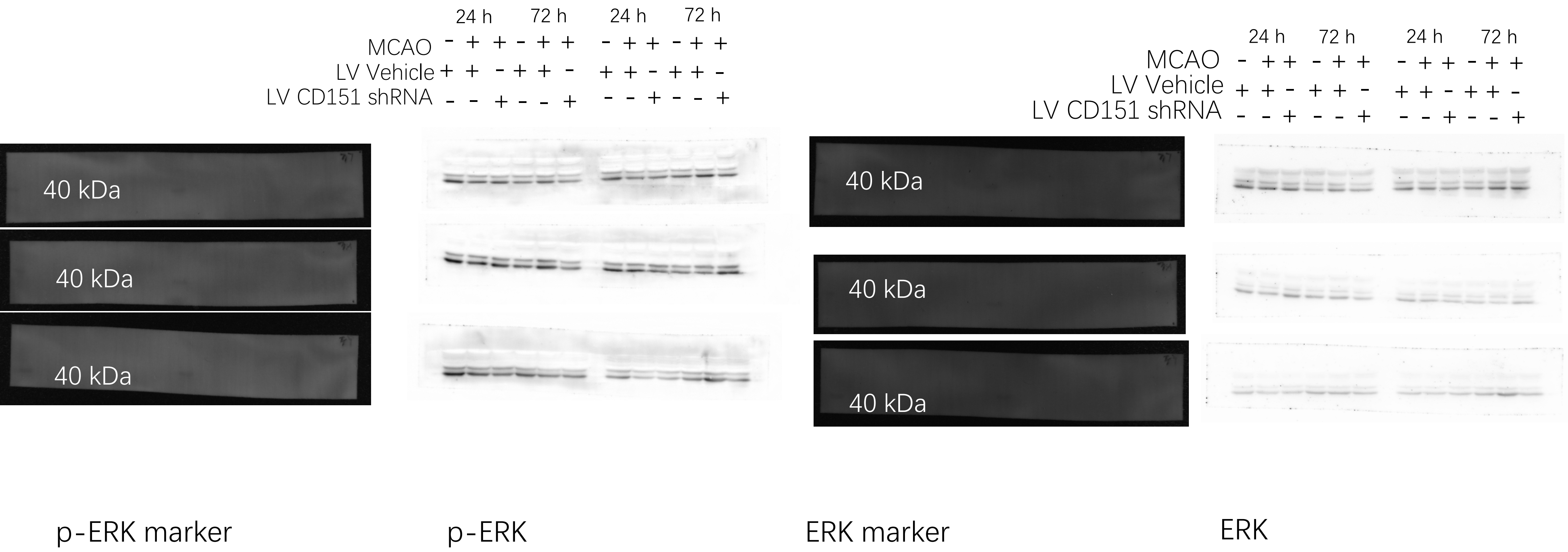
24 h
72 h
24 h
72 h
MCAO - + + - + + - + + - + +
LV Vehicle + + - + + - + + - + + -
LV CD151 shRNA - - + - - + - - + - - +
40 kDa
40 kDa
40 kDa
40 kDa
40 kDa
40 kDa
p-ERK marker
p-ERK
ERK marker
ERK

Fig 5c

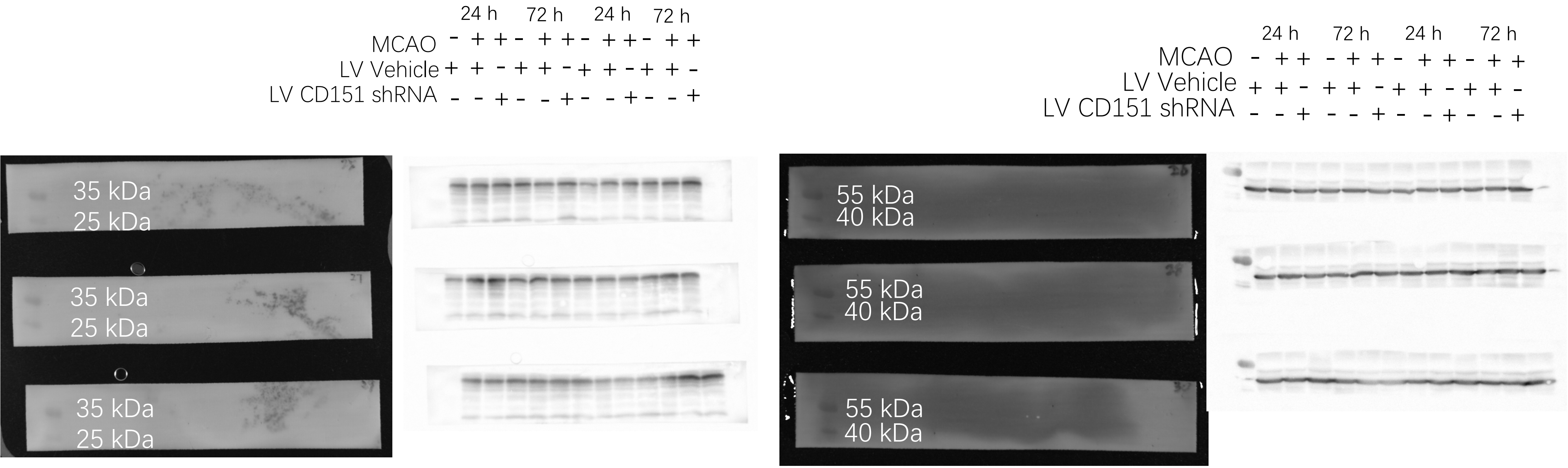


IkB α marker

IkB α

β-actin marker

β-actin

Fig 5d

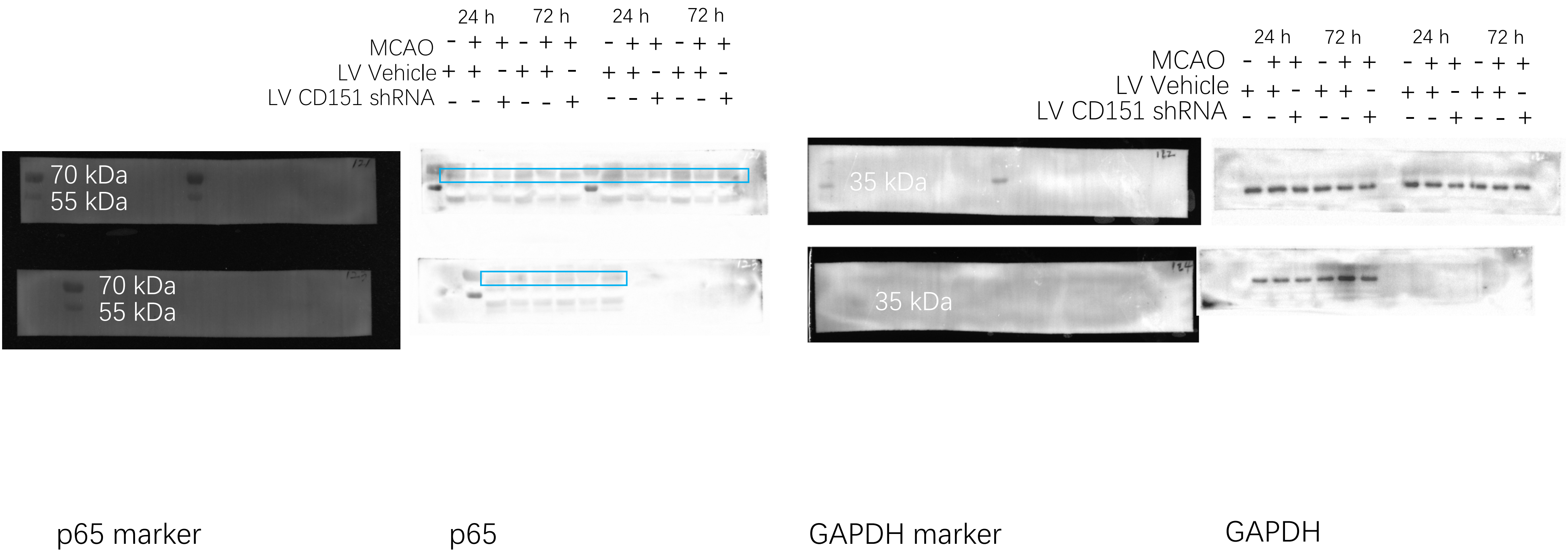
24 h
72 h
24 h
72 h
MCAO - + + - + + - + + - + +
LV Vehicle + + - + + - + + - + + -
LV CD151 shRNA - - + - - + - - + - - +
70 kDa
55 kDa
70 kDa
55 kDa
35 kDa
35 kDa
p65 marker
p65
GAPDH marker
GAPDH

Fig 5e

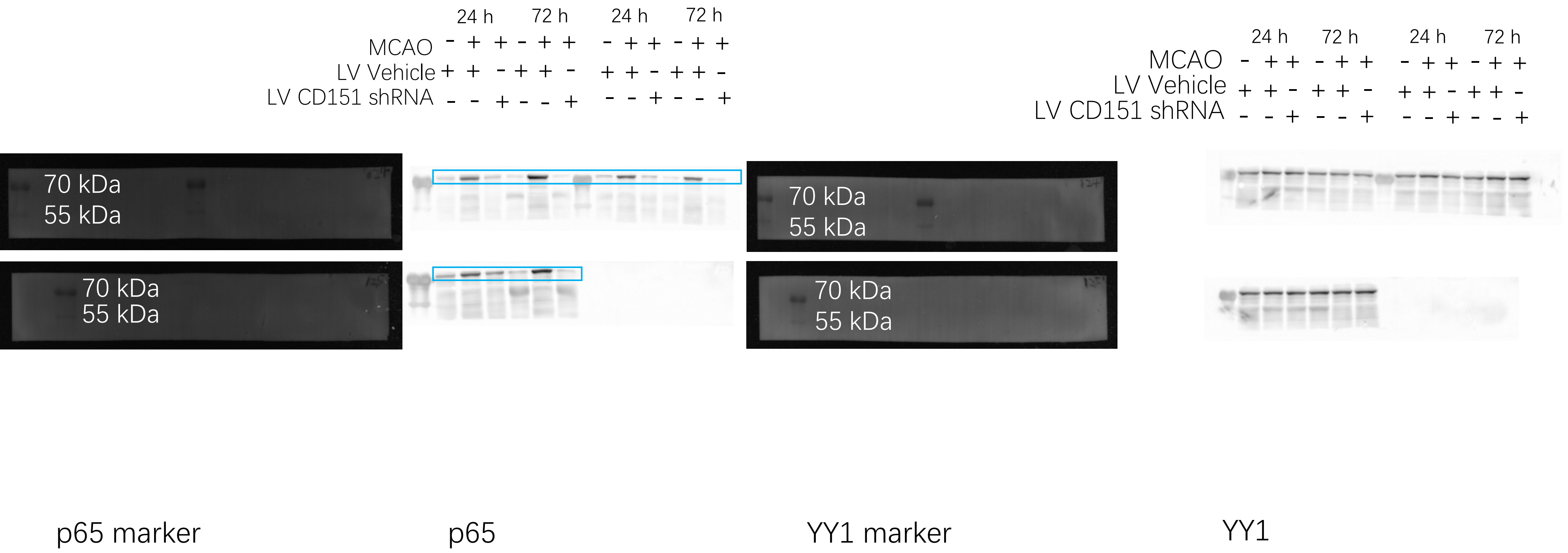
24 h
72 h
24 h
72 h
MCAO - + + - + + - + + - + +
LV Vehicle + + - + + - + + - + + -
LV CD151 shRNA - - + - - + - - + - - +
70 kDa
55 kDa
70 kDa
55 kDa
70 kDa
55 kDa
70 kDa
55 kDa
p65 marker
p65
YY1 marker
YY1

Fig 5f

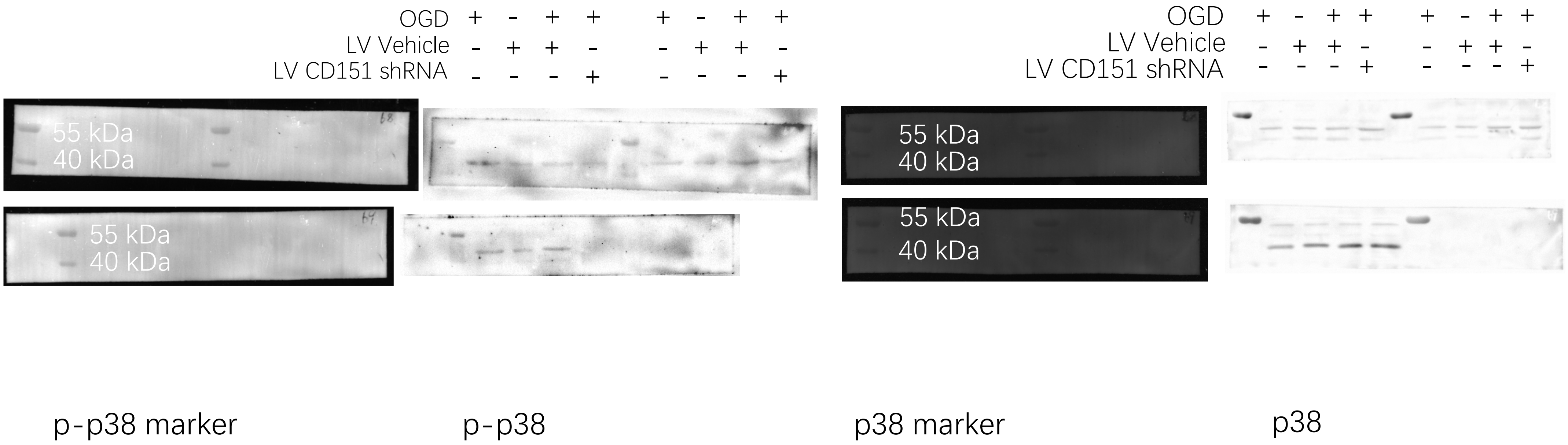
OGD + - + + + - + +
LV Vehicle - + + - - + + -
LV CD151 shRNA - - - + - - - +
55 kDa
40 kDa
55 kDa
40 kDa
OGD + - + + + - + +
LV Vehicle - + + - - + + -
LV CD151 shRNA - - - + - - - +
55 kDa
40 kDa
55 kDa
40 kDa
p-p38 marker
p-p38
p38 marker
p38

Fig 5g

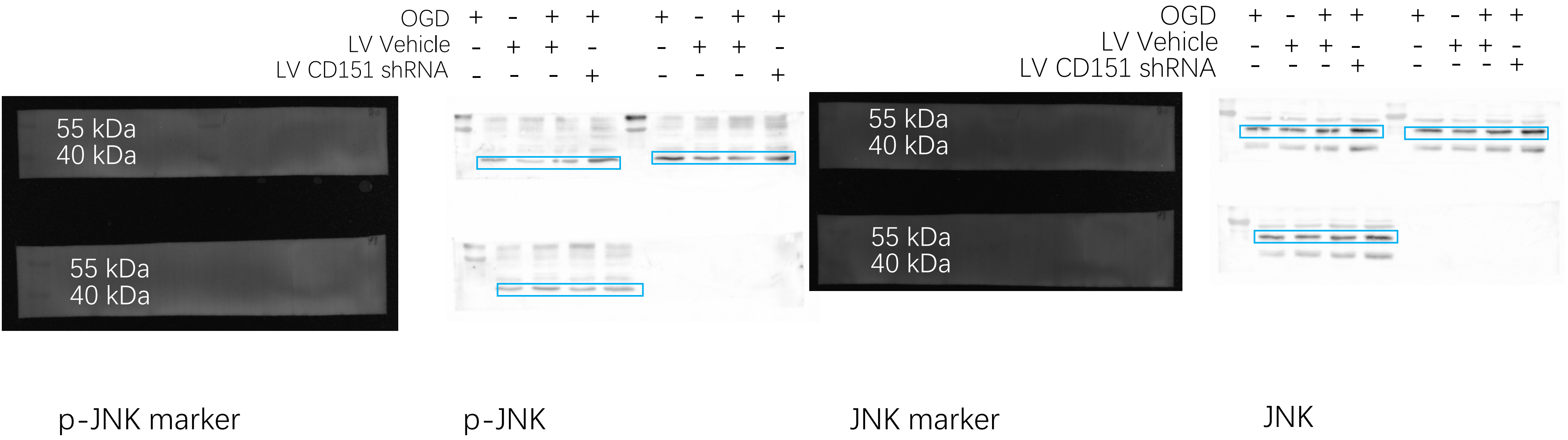
OGD + - + + + - + +
LV Vehicle - + + - - + + -
LV CD151 shRNA - - - + - - - +
55 kDa
40 kDa
55 kDa
40 kDa
55 kDa
40 kDa
55 kDa
40 kDa
p-JNK marker
p-JNK
JNK marker
JNK

Fig 5h

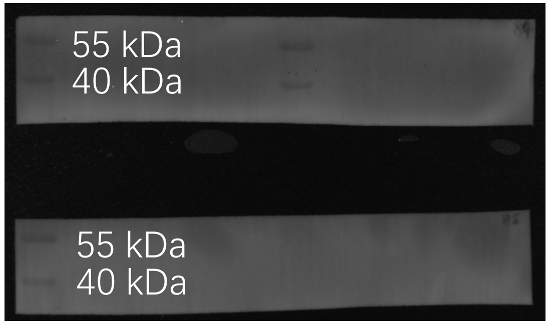


p-ERK marker

| OGD | + | - | + | + | + | - | + | + |
|---|---|---|---|---|---|---|---|---|
| LV Vehicle | - | + | + | - | - | + | + | - |
| LV CD151 shRNA | - | - | - | + | - | - | - | + |

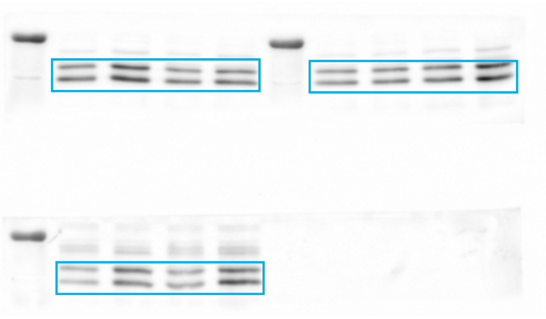

p-ERK

ERK marker
same as p-ERK marker

| OGD | + | - | + | + | + | - | + | + |
|---|---|---|---|---|---|---|---|---|
| LV Vehicle | - | + | + | - | - | + | + | - |
| LV CD151 shRNA | - | - | - | + | - | - | - | + |

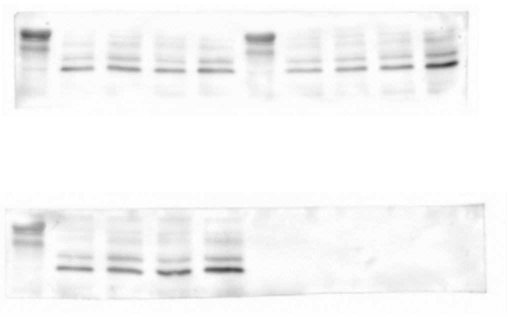

ERK

# Fig 5i

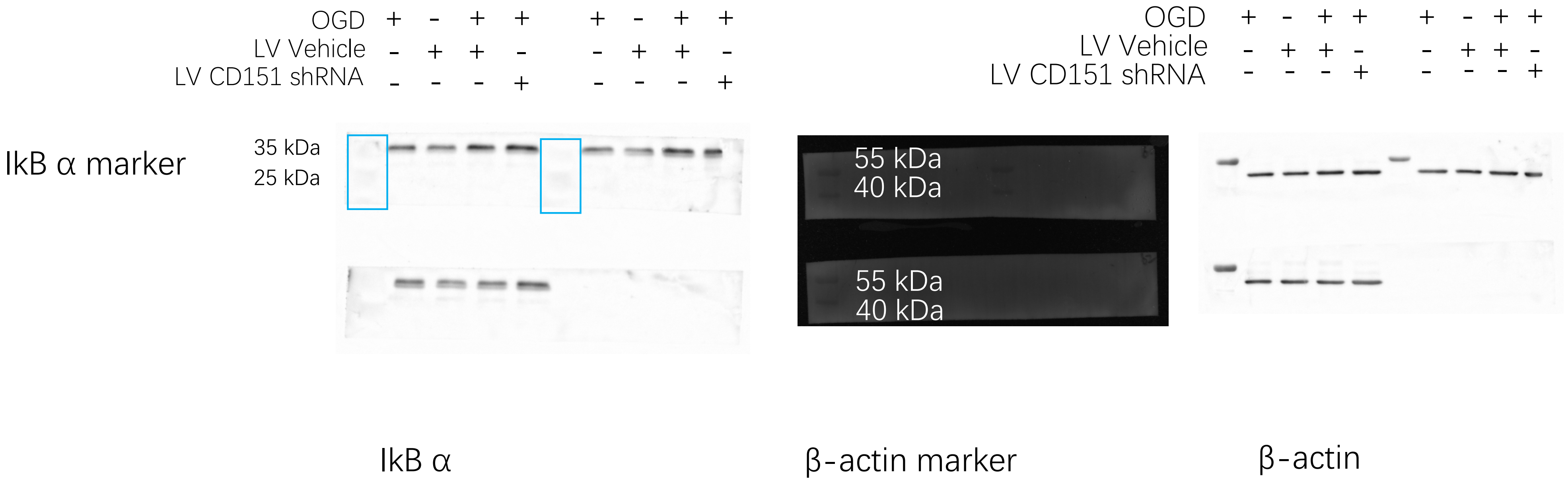
OGD + - + + + - + +
LV Vehicle - + + - - + + -
LV CD151 shRNA - - - + - - - +
IkB α marker
35 kDa
25 kDa
IkB α
55 kDa
40 kDa
55 kDa
40 kDa
β-actin marker
OGD + - + + + - + +
LV Vehicle - + + - - + + -
LV CD151 shRNA - - - + - - - +
β-actin

Fig 5j

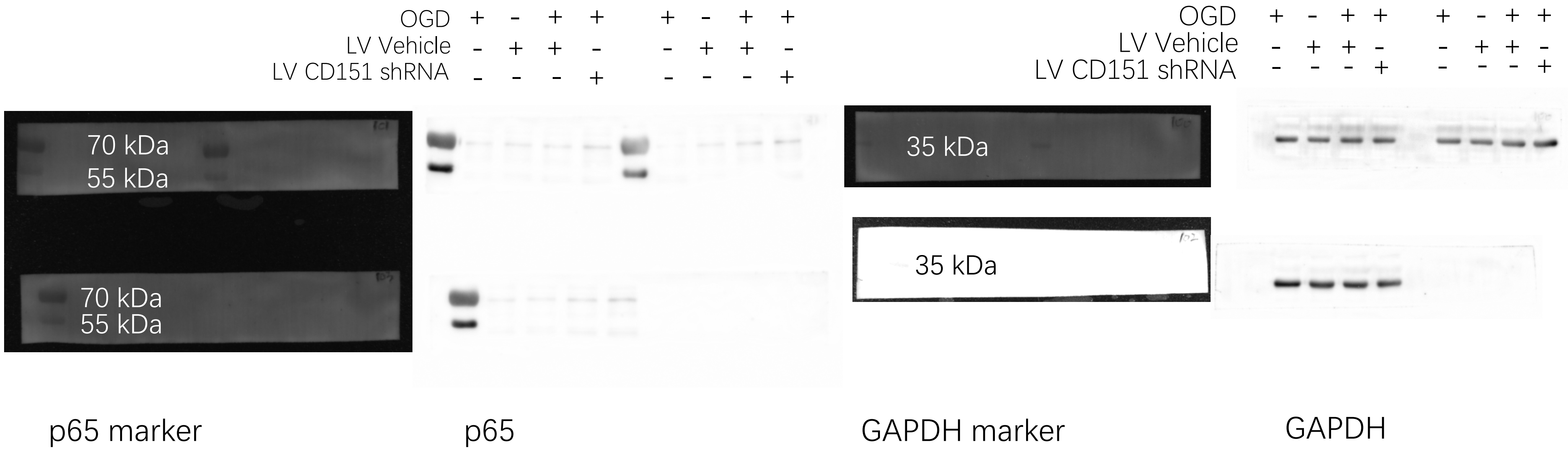
OGD + - + + + - + +
LV Vehicle - + + - - + + -
LV CD151 shRNA - - - + - - - +
70 kDa
55 kDa
70 kDa
55 kDa
p65 marker
p65
OGD + - + + + - + +
LV Vehicle - + + - - + + -
LV CD151 shRNA - - - + - - - +
35 kDa
35 kDa
GAPDH marker
GAPDH

Fig 5k

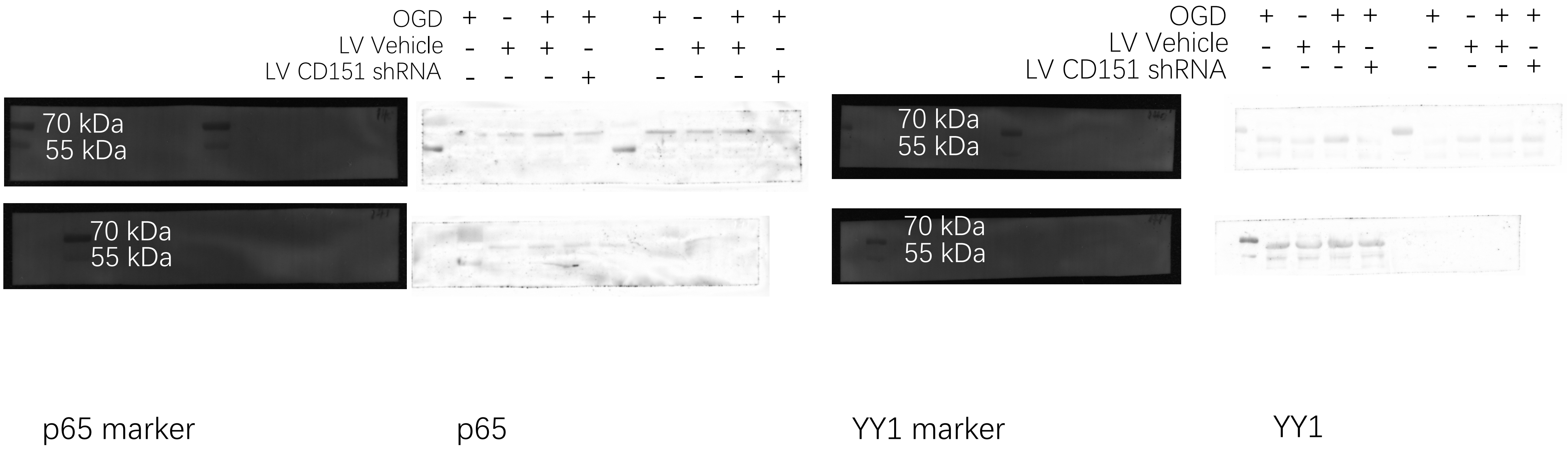
OGD + - + + + - + +
LV Vehicle - + + - - + + -
LV CD151 shRNA - - - + - - - +
70 kDa
55 kDa
70 kDa
55 kDa
p65 marker
p65
OGD + - + + + - + +
LV Vehicle - + + - - + + -
LV CD151 shRNA - - - + - - - +
70 kDa
55 kDa
70 kDa
55 kDa
YY1 marker
YY1

Fig 5l
