# Supplementary material for: Downregulation of CD151 restricts VCAM-1 mediated leukocyte infiltration to reduce neurobiological injuries after experimental stroke
Source: J Neuroinflammation. 2021 May 22;18:118. doi: 10.1186/s12974-021-02171-6 (PMC8140507; doi:10.1186/s12974-021-02171-6)
Supplement: Supplementary file 1 — Additional file 1: Supplementary method. RNA extraction and reverse transcriptase-polymerase chain reaction (RT-PCR). Table S1. Detailed information of lentivirus CD151 shRNA sequences. Figure S1. Different lentivirus CD151 shRNA transfection effectiveness in vitro. Figure S2. Lentivirus CD151 shRNA transfection effectiveness assessment in vivo. Figure S3. Lentivirus transfection effectiveness in vitro. Figure S4. CD151 expression assessment at observation time points in vivo and in vitro. Figure S5. p38 and NF-κB activation were restrained in vivo after CD151 knockdown. The MAPK kinase (i.e., p38, JNK, and ERK) activation was evaluated (a, b, c) using infarcted hemisphere (n = 6 per group) or enriched endothelial cells (g, h) from infarcted hemisphere (n = 3 per group), * and **vs. LV Vehicle + MCAO indicate p < 0.05 and 0.01, respectively. The NF-κB pathway activation evaluated using the (d) IκB α degeneration and the p65 translocation from the (e) cytoplasm to the (f) nucleus in infarcted hemisphere (n = 6 per group), *, ** and *** vs. LV Vehicle + MCAO indicate p < 0.05, 0.01 and 0.001, respectively. Figure S6. Anisomycin increased the phosphorylation of both p38 and JNK in BMVECs. Cultured primary BMVECs were treated with 1μM anisomycin for 3 h (n = 3 per group). An increase in the phosphorylation of p38 (a) and JNK (b) were observed, ** vs. control group indicate p < 0.01. [file 12974_2021_2171_MOESM1_ESM.zip › Western Blot raw data Sup Figure 4.pdf]

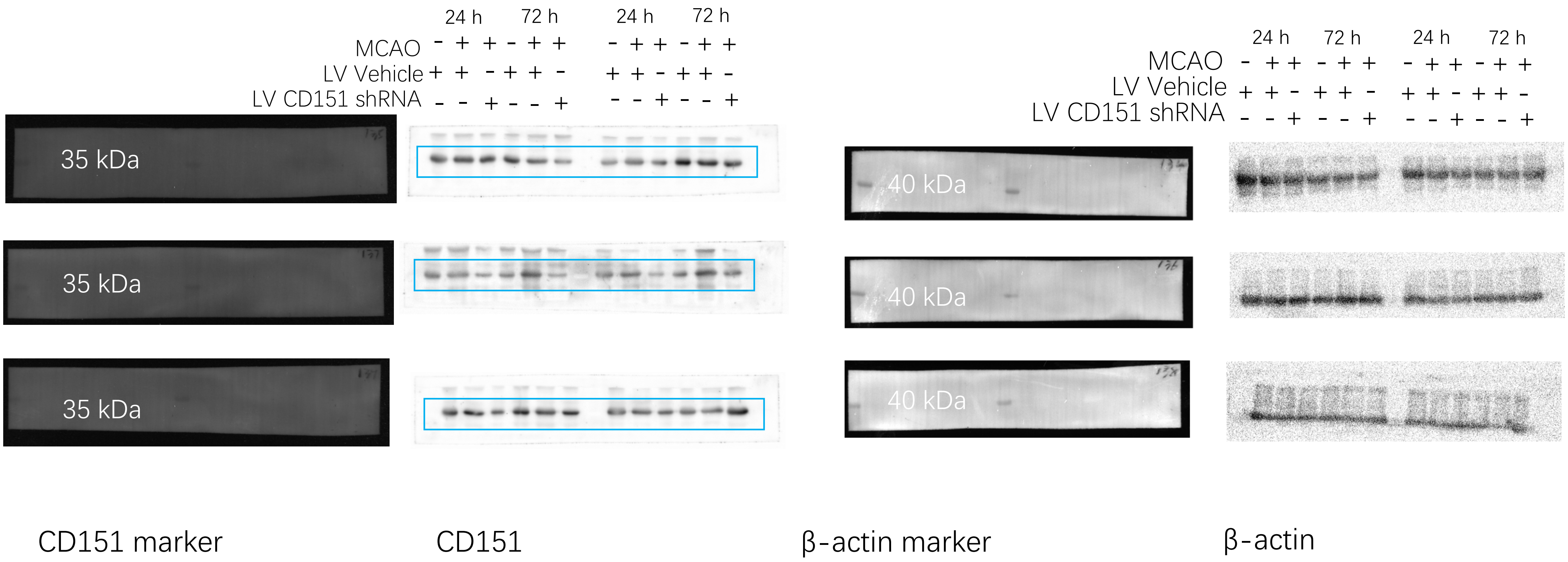
24 h
72 h
MCAO
LV Vehicle
LV CD151 shRNA
35 kDa
40 kDa
CD151 marker
CD151
β-actin marker
β-actin

Sup Fig 4a

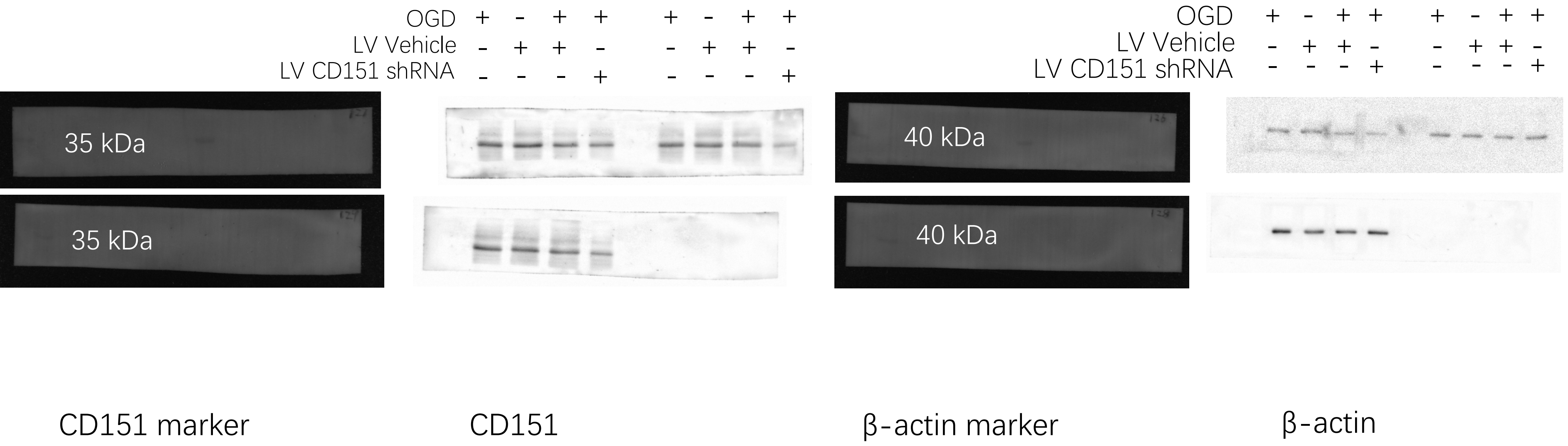
OGD + - + + + - + +
LV Vehicle - + + - - + + -
LV CD151 shRNA - - - + - - - +
35 kDa
35 kDa
40 kDa
40 kDa
OGD + - + + + - + +
LV Vehicle - + + - - + + -
LV CD151 shRNA - - - + - - - +
CD151 marker
CD151
β-actin marker
β-actin

Sup Fig 4b

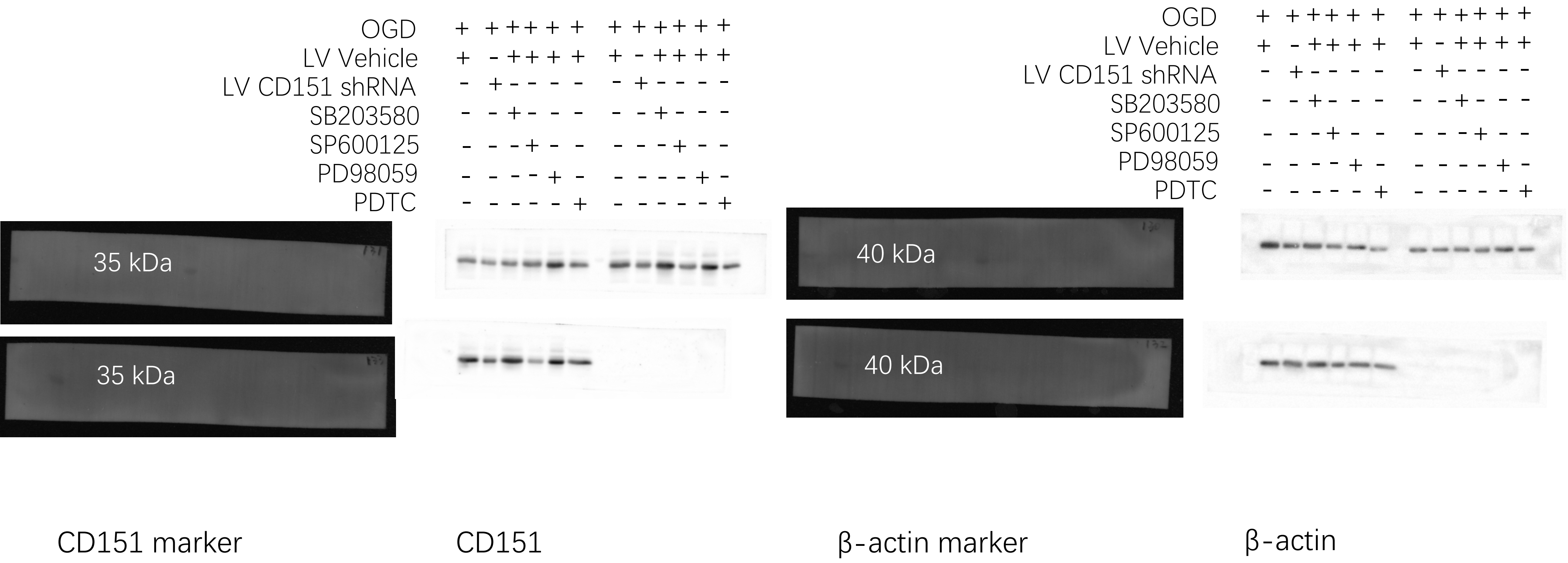

OGD
LV Vehicle
LV CD151 shRNA
SB203580
SP600125
PD98059
PDTC
35 kDa
35 kDa
40 kDa
40 kDa
CD151 marker
CD151
β-actin marker
β-actin

Sup Fig 4c
